# Feasibility indicators in obesity-related behavioral intervention preliminary studies: a historical scoping review

**DOI:** 10.1186/s40814-023-01270-w

**Published:** 2023-03-22

**Authors:** Christopher D. Pfledderer, Lauren von Klinggraeff, Sarah Burkart, Luke Wolfenden, John P. A. Ioannidis, Michael W. Beets

**Affiliations:** 1 Arnold School of Public Health, University of South Carolina, 921 Assembly Street, Columbia, SC 29205 USA; 2 School of Medicine and Public Health, Faculty of Health and Medicine, University of Newcastle, Newcastle, NSW 2318 Australia; 3 Australia and Hunter New England Population Health, Locked Bag 10, Hunter New England Local Health District, Wallsend, NSW 2287 Australia; 4 Departments of Medicine, of Epidemiology and Population Health, of Biomedical Data Science, and of Statistics, and Meta-Research Innovation Center at Stanford (METRICS), Stanford University, Stanford, CA USA

**Keywords:** Pilot, Feasibility, Behavioral, Implementation, Translational science

## Abstract

**Background:**

Behavioral interventions are often complex, operate at multiple levels, across settings, and employ a range of behavior change techniques. Collecting and reporting key indicators of initial trial and intervention feasibility is essential to decisions for progressing to larger-scale trials. The extent of reporting on feasibility indicators and how this may have changed over time is unknown. The aims of this study were to (1) conduct a historical scoping review of the reporting of feasibility indicators in behavioral pilot/feasibility studies related to obesity published through 2020, and (2) describe trends in the amount and type of feasibility indicators reported in studies published across three time periods: 1982–2006, 2011–2013, and 2018–2020.

**Methods:**

A search of online databases (PubMed, Embase, EBSCOhost, Web of Science) for health behavior pilot/feasibility studies related to obesity published up to 12/31/2020 was conducted and a random sample of 600 studies, 200 from each of the three timepoints (1982–2006, 2011–2013, and 2018–2020), was included in this review. The presence/absence of feasibility indicators, including recruitment, retention, participant acceptability, attendance, compliance, and fidelity, were identified/coded for each study. Univariate logistic regression models were employed to assess changes in the reporting of feasibility indicators across time.

**Results:**

A total of 16,365 unique articles were identified of which 6873 of these were reviewed to arrive at the final sample of 600 studies. For the total sample, 428 (71.3%) studies provided recruitment information, 595 (99.2%) provided retention information, 219 (36.5%) reported quantitative acceptability outcomes, 157 (26.2%) reported qualitative acceptability outcomes, 199 (33.2%) reported attendance, 187 (31.2%) reported participant compliance, 23 (3.8%) reported cost information, and 85 (14.2%) reported treatment fidelity outcomes. When compared to the Early Group (1982–2006), studies in the Late Group (2018–2020) were more likely to report recruitment information (OR=1.60, 95%CI 1.03–2.49), acceptability-related quantitative (OR=2.68, 95%CI 1.76–4.08) and qualitative (OR=2.32, 95%CI 1.48–3.65) outcomes, compliance outcomes (OR=2.29, 95%CI 1.49–3.52), and fidelity outcomes (OR=2.13, 95%CI 1.21, 3.77).

**Conclusion:**

The reporting of feasibility indicators within behavioral pilot/feasibility studies has improved across time, but key aspects of feasibility, such as fidelity, are still not reported in the majority of studies. Given the importance of behavioral intervention pilot/feasibility studies in the translational science spectrum, there is a need for improving the reporting of feasibility indicators.

**Supplementary Information:**

The online version contains supplementary material available at 10.1186/s40814-023-01270-w.

## Background

Pilot/feasibility (aka proof-of-concept, exploratory trial, preliminary study, evidentiary, vanguard) studies play an essential role in the process of conducting larger-scale clinical trials by providing information about the potential efficacy and feasibility of an intervention [1] and addressing uncertainties around conducting a larger-scale study [2–7]. This is evidenced by the importance funding agents place on conducting pilot/feasibility studies, such as the National Institutes of Health (NIH) and the Medical Research Council (MRC), where multiple mechanisms (e.g., R34, R01 small pilot studies from the NIDDK, R21, R03, National Prevention Research Initiative) provide financial support for conducting early-stage, preliminary studies.

Key information collected during pilot/feasibility studies includes *trial feasibility*—can we recruit and retain the target population; *intervention feasibility*—can we deliver the intervention and do participants like it; and *preliminary efficacy—*does the intervention show a preliminary signal of promise [8–10]. Of these, trial- and intervention-feasibility metrics have garnered heightened attention over the past decade. Multiple reporting frameworks [10–16] emphasize and reinforce that trial- and intervention-feasibility indicators are essential to determine whether a trial can be successfully conducted, if changes to the design and/or implementation are warranted, and whether participant retainment is high enough so individuals receive a sufficient dosage of the intervention to result in improved outcomes. Funding agencies like the NIH and the MRC make clear that the role of pilot studies is to assess whether an intervention can be done, and such information is essential to making decisions regarding whether one should proceed with a larger-scale trial of an intervention [1, 17].

Undertaking studies that gather information on trial- and intervention-feasibility creates a foundation for the optimization and successful scaling-up to larger-scale trials [11, 12, 18]. In the behavioral sciences, where obesity-related interventions often consist of delivering complex interventions across multiple levels/settings and use a range of behavior change techniques, collecting and reporting key aspects of feasibility during the initial testing of the intervention is of heightened importance. Previous research on behavioral interventions has shown that increasing complexity decreases understanding of how the intervention operates and increases the difficulty of delivering the intervention as intended [19]. By focusing on feasibility during the preliminary stages of obesity-related behavioral intervention development (pilot/feasibility studies), researchers put themselves at lower risk of designing interventions that fail at scale due to a lack of understanding about effective design and/or implementation [18, 20].

Reporting guidelines, frameworks, and translational science models advocate for the conduct of high-quality early-stage pilot/feasibility studies as an important step in developing maximally potent and implementable prevention and treatment interventions, many of which are obesity-related [21–24]. Comprehensive pilot/feasibility studies reporting on key information (*trial* and *intervention feasibility*) provide the best evidence for decision making when scaling up to a larger trial. To date, no review has examined the reporting of feasibility indicators within obesity-related behavioral intervention pilot/feasibility studies. Questions remain as to how the field of obesity-related intervention science reports feasibility indicators from pilot/feasibility studies and to what extent the focus on feasibility indicators has evolved over time. Understanding how the field of obesity-related behavioral science has historically utilized feasibility indicators and how the design and conduct of pilot/feasibility studies has evolved over time is an important perspective to gain in order to optimize preliminary behavioral interventions. The aims of this study, therefore, are to (1) conduct a historical scoping review of the reporting of feasibility indicators in obesity-related behavioral pilot/feasibility studies published up to and including 2020, and (2) describe trends in the amount and type of feasibility indicators reported in obesity-related pilot/feasibility studies published across three time periods that span four decades from 1982 to 2020.

## Methods

This scoping review was conducted and is reported according to the Preferred Reporting Items of Systematic Reviews and Meta-Analyses extension for scoping reviews (PRISMA-ScR) guidelines [25].

### Search strategy

A systematic literature search was conducted in four online databases including PubMed/Medline, Embase/Elsevier, EBSCOhost, and Web of Science in September 2021. A combination of Medical Subject Heading (MeSH), EMTREE, and free-text terms and Boolean operators as appropriate for each database were used to identify eligible publications. Each search included one or more of the following terms to identify pilot studies—pilot, feasibility, preliminary, proof-of-concept, vanguard—and the following terms to identify obesity-related behavioral interventions—obesity, overweight, physical activity, fitness, exercise, diet, nutrition, sedentary, or screen. The following additional filters were applied in databases when available: English language, human species, articles only, and peer-reviewed journals.

### Eligibility criteria

Published pilot studies that employed a behavioral intervention strategy on a topic related to obesity were considered for inclusion in this scoping review. Behavioral interventions were defined as interventions that target actions which lead to improvements in health indicators [26, 27], separate from mechanistic, laboratory, pharmacological, feeding/dietary supplementation, and medical device or surgical procedure studies. Pilot studies were defined as those studies which are conducted separately from and prior to a large-scale trial and are designed to test the feasibility of an intervention and/or provide evidence of preliminary effects before scale-up [3, 21, 28]. Exclusion criteria were articles that only described the development of a pilot study (protocols), studies that employed a non-behavioral intervention strategy (as described above), studies that did not deliver an intervention to participants (observational/cross-sectional), qualitative studies, and dissertations.

### Sampling strategy

Given the broad nature of this review’s search strategy and inclusion criteria, an a priori decision was made to use a multi-stage sampling procedure in order to select a random sample of studies from three distinct time points for comparison. There was an unequal distribution of published studies across all timepoints, with most studies (83.2%) published between 2011 and 2020. Thus, a simple random sample would be unlikely to capture enough articles to sufficiently represent those published in earlier years. Starting with the earliest published citation, titles/abstracts, and full texts were screened in chronological order by year of publication until 200 studies were identified that met the inclusion criteria. This resulted in a collection of studies that spanned from 1982 to 2006. It is important to note that 1982 was the starting point for this scoping review simply because no relevant records pre-1982 were retrieved from the databases. The two additional time points were then determined to be 2011–2013 and 2018–2020, which represents an equal 5-year gap between each successive time point. Studies published between 2011 and 2013 and between 2018 and 2020 were assigned random numbers with STATA’s “rannum” command and screened in order of randomization until an additional 200 studies that met the inclusion criteria were identified from each group [29].

### Power analysis

The final sample size of 600 (200 per group) was based on detecting a minimal difference between the three distinct timepoints in the probability (binary presence/absence) of reporting a feasibility marker. Considering an alpha of 0.05, power analysis revealed that a logistic regression, with the binary variable for a feasibility indicator as the dependent variable and two “dummy variables” representing two time period categories, could detect a minimal difference of an odds ratio of 1.35. This represents a difference in the reporting of a feasibility indicator of 20% of articles versus 26% of articles.

### Screening process

Database search results were electronically downloaded as a RIS file and uploaded to Covidence systematic review software (Veritas Health Innovation, Melbourne, Australia) for review. Duplicate references were identified as the RIS files were uploaded to Covidence and were screened out of the review process. Title and abstract screening were completed in duplicate by two reviewers (CDP and MWB) to identify references that met the eligibility criteria. Disagreements were solved by having a third member (LV) of the research team review the reference and make a final decision. Full-text PDFs were retrieved for references that passed the initial title and abstract screening process and were reviewed in duplicate by three members of the research team (CDP, MWB, and LV).

### Data extraction and coding

#### Study- and participant-level descriptive characteristics

Relevant study-level and participant-level descriptive characteristics were extracted from included studies by five members of the research team and were coded in an Excel spreadsheet. These included characteristics such as study location, publication year, design, treatment length, sample size, age and sex of participants, intervention setting, and behaviors targeted by the intervention. Because of the large amount of data extracted, it was not possible to doubly extract and code each individual study. Instead, the lead author (CDP), another member of the research team (LV), and three research assistants doubly coded a training set of studies until 100% consistency was reached. At that point, individual studies were assigned to members of the research team for a single round of data extraction and coding.

#### Feasibility indicators

Table 1 provides the operational definitions of each feasibility indicator identified for this review and the outcomes extracted and/or calculated. The seven feasibility indicators, which were chosen a priori after a review of reporting guidelines/frameworks related to preliminary studies [10–12, 17, 30] included indicators of *trial feasibility*—recruitment capability and retention, and indicators of *intervention feasibility*—participant acceptability, attendance, compliance, cost, and treatment fidelity. Definitions for each of these indicators were adapted from the NIH [17] and other peer-reviewed sources [11, 12, 30]. Because the terms “feasibility” and “acceptability” are sometimes used synonymously [7], it was important to define individual feasibility indicators a priori. Thus, while the term “acceptability” may have been used to describe other aspects of feasibility (recruitment, retention, etc.), if those aspects were not related to participant acceptability (enjoyment, satisfaction, tolerability, safety, etc.), they were not coded as an acceptability-related indicator but were instead coded based on our definitions of feasibility indicators.

**Table 1:** Operational definitions of trial- and intervention-related feasibility indicators

Category	Indicator	Definition^**a**^	Search terms^**b**^	Outcomes
Trial-Related Feasibility Indicators	Recruitment Capability	The proportion of eligible participants who are enrolled at baseline of the study.	Search was completed manually. Information typically provided in CONSORT diagrams.	Recruitment Capability (%) calculated as: $$\left(\frac{\# participants\ at\ baseline}{\# eligible\ participants}\right)\times 100\%$$
	Retention	The proportion of enrolled participants who are present throughout the full length of the treatment.	Search was completed manually. Information typically provided in CONSORT diagrams.	Retention (%) calculated as: $$\left(\frac{\# participants\ at\ treatment\ end}{\# participants\ at\ baseline}\right)\times 100\%$$
Intervention-Related Feasibility Indicators	Treatment Fidelity^c^	Content, frequency, duration, and coverage as originally intended	adhere*, deliver*	Deliverer adherence to the originally intended treatment plan
	Acceptability	The perception among participants’ that the treatment is agreeable or satisfactory.	accept*, appropriate*, enjoy*, satisf*, fun, safety, difficult*, tolera*	Participant-rated acceptability, satisfaction, enjoyment, perceptions of safety, difficulty, tolerability of treatment, number of adverse events
	Attendance^c^	The proportion of total sessions offered to participants to the actual number of sessions participants attended.	attend*, engag*,	Attendance Rate (%) calculated as: $$\left(\frac{\# sessions\ participants\ attend}{\# sessions\ offered}\right)\times 100\%$$
	Compliance^c^	Participants’ level of adherence to the content, frequency, duration, and coverage of the treatment as delivered by the research team.	comply, complian*, adhere*	Percentage of participants who complied with intervention procedures; Number of adaptations made by participants
	Cost	Monetary costs associated with delivering the intervention to participants.	cost*, economic eval*, $, AUD, CAD, CHF, £, pound sterling, €, euro, ¥, yen	Monetary costs associated with the intervention

^a^Definitions adapted from the National Institutes of Health [17] and other peer-reviewed sources [11, 12, 30]

^b^Search terms are those used for text mining procedures

^c^These terms are common markers of implementation fidelity [31]

Data extracted for each feasibility indicator included descriptions of the types of feasibility indicators being measured and any reported quantitative outcomes related to those measures. The presence of any qualitative feasibility indicators was also cataloged, including participant interviews, open-ended survey response questions about intervention acceptability, and mixed methodology related to participant acceptability. Each of these variables was classified as either “present” or “absent”. Other variables of interest included the reporting of any funding, mentioning feasibility-related parameters in the purpose statement, citing any guidelines or frameworks related to the reporting of preliminary studies, and reporting/conducting any statistical tests to determine preliminary efficacy. When studies had cited a separate process evaluation or other publication related to feasibility of the original pilot study, those cited publications were also searched for the reporting of feasibility indicators.

The identification of feasibility indicators and the other variables of interest as reported in pilot studies was done by utilizing a combination of text mining [32] and manual search procedures. Text mining procedures were conducted in NVivo 12 Plus Qualitative Data Analysis Software (QSR International, 2021) by three members of the research team and consisted of full-text searches with keywords related to feasibility outcomes. A full list of keywords was manually created by training with a randomized sample of 50 pilot study articles and scanning full-text articles for keywords relate to feasibility until saturation had been achieved. Once the presence of feasibility outcomes had been detected in all pilot studies with text mining procedures, manual extraction of specific feasibility outcome-related information was completed by three members of the research team. Full-text PDFs were also manually searched to ensure text mining procedures identified all possible reported feasibility indicators.

### Statistical analysis

Descriptive statistics were compared between each of the three time periods using Kruskal-Wallis tests and chi-square tests when appropriate. These tests were also conducted to ensure the random sampling procedure used to select studies did not produce any systematic differences between groups. A series of univariate logistic regression models were employed to assess changes in the reporting of feasibility indicators across time. The presence or absence of each feasibility indicator was treated as the binary dependent variable while two dummy variables representing time periods 2011–2013 and 2018–2020 (with 1982–2006 as the reference category) were independent variables. Predicted marginal probabilities were also calculated with STATA’s “margins” command to determine the probability of reporting each feasibility indicator at each time period, holding all other variables at their means. Finally, hierarchical Poisson regression models were employed to assess the associations between the quantity of feasibility indicators reported and reporting funding, mentioning feasibility-related parameters in the purpose statement, and citing any guidelines/frameworks related to the reporting of preliminary studies. The presence or absence of funding, mentioning feasibility-related parameters in the purpose statement, and citing guidelines/frameworks were treated as binary independent variables while the number of feasibility indicators reported was treated as the dependent variable. A hierarchical predictor variable entry method was employed to examine the independent association of funding, mentioning feasibility in the purpose statement, and citing guidelines/frameworks (Model 1) and the statistical control of time period (Model 2). The *n* alpha level of *p* < 0.05 was considered suggestive and *p* <0.005 was considered formally statistically significant. Analyses were carried out using STATA v17.0 statistical software package (College Station, Texas, USA).

## Results

### Search results

**Fig. 1:**
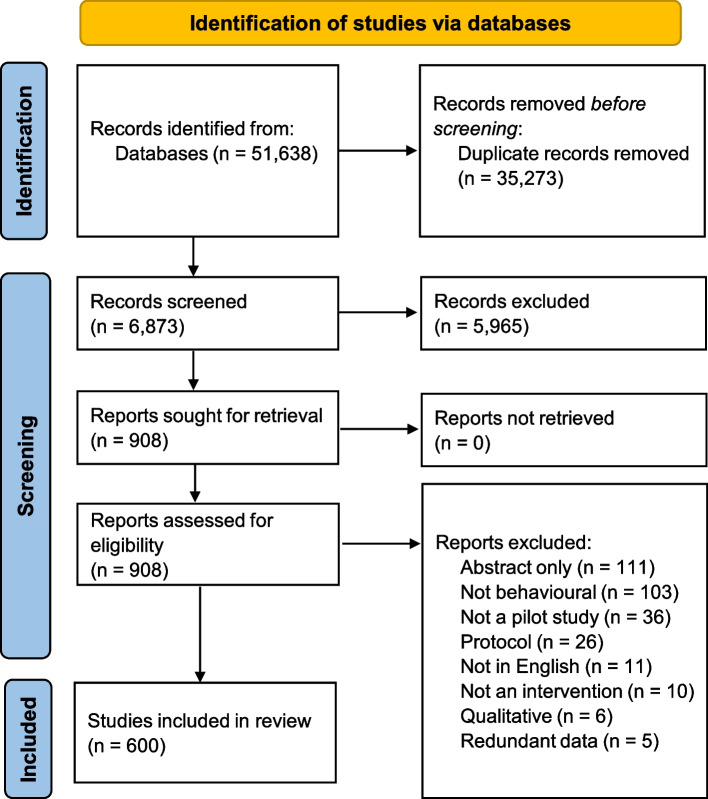
Preferred Reporting Items for Systematic Reviews and Meta-Analyses (PRISMA) consort diagram

Figure 1 displays the PRISMA consort diagram, which communicates the screening process. A total of 51,638 citations were identified across databases. After duplicates were removed, 16,365 citations remained and 6873 of those citations were screened. Due to the multi-stage randomization procedure for selecting studies (see “Screening process” section) a total of 9492 of the 16,365 articles were never screened. Full-text screening was done until we obtained 200 eligible articles from each time period, which resulted in 600 articles being included in this review. For the 1982–2006 time period, 310 full-text articles were screened, while a total of 350 and 248 full-text articles were screened for the 2011–2013 and the 2018–2020 time periods, respectively. The supplementary file contains a reference list of all included studies.

### Descriptive characteristics of included studies

Study- and participant-level descriptive characteristics are reported in Table 2. This information is reported for all studies and is also reported separately for studies in each time period category. Most studies were conducted in North America (*n*=399, 66.5%), were a RCT (299, 49.8%), had two arms (*n*=326, 54.3%), measured outcomes at two timepoints (*n*=428, 71.3%), had adult participants (*n*=427, 71.7%), and included both male and female participants (*n*=434, 72.3%). The median treatment length was 12 weeks (IQR = 8–26 weeks), and the median baseline sample size was 48 participants (IQR = 28–89 participants). The mean age of youth participants was 10.9 ± 3.5 years, and the mean age of adult participants was 48.6 ± 15.1 years.

**Table 2:** Characteristics of included studies (*N=*600)

Characteristics	All studies (1982–2020)		Early Group (1982–2006)		Middle Group (2011–2013)		Late Group (2018–2020)	
	*N* = 600		*N* = 200		*N* = 200		*N* = 200	
	*N*	Percent (%)	*N*	Percent (%)	*N*	Percent (%)	*N*	Percent (%)
**Location**								
North America	399	66.5	151	75.5	138	69.0	110	55.0
Europe	122	20.3	26	13	32	16.0	64	32.0
Australia	43	7.2	16	8	19	9.5	8	4.0
Asia	25	4.2	4	2	8	4.0	13	6.5
South America	7	1.2	2	1	1	0.5	4	2.0
Africa	4	0.7	1	0.5	2	1.0	1	0.5
**Design**								
Randomized controlled trial (RCT)	299	49.8	108	54.0	90	45.0	101	50.5
Non-randomized, single group	223	37.2	66	33.0	85	42.5	72	36.0
Non-randomized, multi-intervention	45	7.5	15	7.5	15	7.5	15	7.5
Randomized, multi-intervention	33	5.5	11	5.5	10	5.0	12	6.0
**Number of arms**								
1	235	39.2	74	37.0	86	43.0	75	37.5
2	326	54.3	109	54.5	106	53.0	111	55.5
3	29	4.8	13	6.5	6	3.0	10	5.0
4	9	1.5	14	7.0	2	1.0	3	1.5
5	1	0.2	0	0.0	0	0.0	1	0.5
**Number of measurement timepoints**								
1	5	0.8	2	1.0	0	0.0	3	1.5
2	428	71.3	154	77.0	143	71.5	131	65.5
3	148	24.7	38	19.0	49	24.5	61	30.5
4	19	3.2	6	3.0	8	4.0	5	2.5
**Treatment length**								
6 weeks or less	140	23.3	45	22.5	45	22.5	50	25.0
7–12 weeks	236	39.3	73	36.5	75	37.5	88	44.0
13–26 weeks	150	25.0	46	23.0	59	29.5	45	22.5
27–52 weeks	55	9.2	25	12.5	17	8.5	13	6.5
53 weeks or more	19	3.2	11	5.5	4	2.0	4	2.0
**Baseline sample size**								
1–24	124	20.7	45	22.5	34	17.0	44	22.0
25–49	183	30.5	57	28.5	57	28.5	69	34.5
50–99	165	27.5	44	22.0	65	32.5	56	28.0
100 or more	128	21.3	54	27.0	44	22.0	31	15.5
**Age of participants**								
Youth (0–18 years)	173	28.3	46	23.0	69	34.5	58	29.0
Adult (18+ years)	427	71.7	154	77.0	131	65.5	142	71.0
**Sex of participants**								
Women only	139	23.2	63	31.5	41	20.5	35	17.5
Men only	27	4.5	7	3.5	10	5.0	10	5.5
Both	434	72.3	130	65.0	149	74.5	155	77.5
**Intervention setting**								
Clinic	154	25.7	64	32	50	25	40	20
Community	122	20.3	50	25	36	18	36	18
Remote	99	16.5	18	9	37	18.5	44	22
K-12 school	96	16	26	13	37	18.5	33	16.5
Home	54	9	18	9	21	10.5	15	7.5
Workplace	32	5.3	6	3	12	6	14	7
University	17	2.8	6	3	4	2	7	3.5
Care Facility (e.g., nursing home)	15	2.5	9	4.5	1	0.5	5	2.5
Church	10	1.7	3	1.5	2	1	5	2.5
Prison	1	0.2	0	0	0	0	1	0.5
**Target behavior**^**a**^								
Physical activity	465	77.5	139	69.5	165	82.5	161	80.5
Nutrition	347	57.8	125	62.5	119	59.5	103	51.5
Mental health	57	9.5	21	10.5	13	6.5	23	11.5
Sleep	37	6.2	7	3.5	7	3.5	23	11.5
Screen time	23	3.8	5	2.5	7	3.5	11	5.5
Tobacco	22	3.7	13	6.5	6	3	3	1.5
Alcohol	7	1.2	3	1.5	3	1.5	1	0.5
STIs	4	0.7	1	0.5	0	0	3	1.5
**Funding**^**a**^								
Any funding	402	67	112	56	134	67	156	78
NIH (USA)	142	35.3	40	35.7	48	35.8	54	34.6
NHMRC (Australia)	13	3.2	1	0.8	5	3.7	7	4.5
NIHR (Europe)	11	2.7	0	0	0	0	11	7.1
MRC (UK)	8	2.0	0	0	4	2.9	4	2.6
CIHR (Canada)	7	1.7	3	2.7	1	0.7	3	1.9
Other government	78	19.4	22	19.6	31	23.1	25	16.0
Foundation/Center	118	29.4	40	35.7	39	29.1	39	25
University/Institution	55	13.7	15	13.4	15	11.1	25	16
Early career awards	33	8.2	6	5.4	10	7.5	17	10.9
Doctoral	19	4.5	4	3.6	8	5.9	7	4.5
Private/Corporation	17	4.2	10	8.9	2	1.5	5	3.2
Post-doctoral	15	3.7	5	4.5	4	2.9	6	3.8

^a^Sums exceed 600 as studies could qualify for multiple categories

Many studies were conducted in clinic (*n*=154, 25.7%) or community settings [e.g., YMCAs, Boys and Girls Clubs, parks and recreation facilities, free-living environments] (*n*=122, 20.3%), although settings such as remote-delivery, K-12 schools, homes, workplaces, universities, care facilities, churches, and prisons were also represented. Physical activity (*n*=465, 77.5%) and nutrition (*n*=347, 57.8%) were the most represented target behaviors, with many studies targeting both behaviors (*n*=239, 39.8%).

### Purpose statements, framework usage, funding, and preliminary efficacy

A total of 357 (59.5%) studies mentioned feasibility-related parameters in the purpose statement, 58 (9.7%) cited a guideline/framework for the reporting of preliminary studies, and 402 (67.0%) reported a funding source of any kind. The most commonly cited guidelines/frameworks included the Medical Research Council guidance [1] (*n*=17, 29.3%), the CONSORT extension for pilot and feasibility studies [10] (*n=*15, 25.9%), and the RE-AIM framework [33] (*n=*12, 20.7%). The most commonly cited funding sources were the NIH (*n=*142, 35.3%) and foundation/center grants (*n=*118, 29.4%). Significant differences in study characteristics between the three time periods were only found in the reporting of funding, which increased with each later year category, *X*^2^ (2, *N=*600) = 14.5, *p*<0.001. Conducting/reporting statistical analyses related to preliminary efficacy was identified in 561 (93.5%) studies. Of studies that conducted/reported statistical analyses related to preliminary efficacy, 371 (66.1%) made statements about preliminary efficacy in the conclusion. Of these studies, 298 (80.3%) made positive statements about the preliminary efficacy of the intervention.

### Reporting of feasibility indicators

Table 3 provides the number and percentage of articles reporting each feasibility indicator for the total sample and across time periods.

**Table 3:** Presence or absence of the reporting of feasibility indicators across time

Feasibility indicators	All studies (1982–2020)		Early Group (1982–2006)		Middle Group (2011–2013)		Late Group (2018–2020)	
	*N* = 600		*N* = 200		*N* = 200		*N* = 200	
	*N*	Percent (%)	*N*	Percent (%)	*N*	Percent (%)	*N*	Percent (%)
**Recruitment**	428	71.3	134	67	141	70.5	153	76.5
**Retention**	595	99.2	197	98.5	198	99	200	100
**Acceptability**								
Description of quantitative Measure	192	32	43	21.5	58	29	91	45.5
Quantitative outcome	219	36.5	52	26	70	35	97	48.5
Description of qualitative outcome	143	23.8	29	14.5	42	21	72	36
Qualitative outcome	157	26.2	39	19.5	46	23	72	36
**Attendance**								
Description of measure	109	18.2	31	15.5	35	17.5	43	21.5
Outcome	199	33.2	65	32.5	71	35.5	63	31.5
**Compliance**								
Description of measure	162	27	41	20.5	52	26	69	34.5
Outcome	187	31.2	48	24	55	27.5	84	42
**Cost**	23	3.8	8	4.0	5	2.5	10	5.0
**Treatment fidelity**								
Description of measure	109	18.2	22	11	39	19.5	48	24
Outcome	85	14.2	21	10.5	24	12	40	20
**Total outcomes reported**								
1	65	13	24	12	29	14.5	12	6
2	143	27	61	30.5	44	22	38	19
3	168	29.2	65	32.5	56	28	47	23.5
4	136	21.3	39	19.5	43	21.5	54	27
5	58	6.5	7	3.5	20	10	31	15.5
6	29	2.7	4	2	8	4	17	8.5
7	1	0.3	0	0	0	0	1	0.5
Mentioned feasibility-related parameters in the purpose statement	357	59.5	100	50	116	58	141	70.5
Cited a guideline/framework for the reporting of preliminary studies	58	9.7	2	1	11	5.5	45	22.5
Conducted/reported statistical analyses related to preliminary efficacy	561	93.5	186	93	192	96	183	91.5
Made any statements about preliminary efficacy in conclusion	371	61.8	101	50.5	128	64	142	71
Made positive statements about preliminary efficacy in conclusion.	298	49.7	90	45	99	49.5	109	54.5

### Trial-related feasibility indicators

For the total sample, 428 (71.3%) studies provided information necessary to calculate recruitment rates and 595 (99.2%) provided information necessary to calculate retention rates. The mean recruitment rate was 69.6 ± 29.1% (median = 76.3, IQR = 48.8–95.4) and the mean retention rate was 83.6 ± 18.8% (median = 89.7, IQR = 75–100).

### Intervention-related feasibility indicators

For the total sample, 192 (32%) included a description of a quantitative measure of acceptability, 219 (36.5%) reported a quantitative outcome related to acceptability, 143 (23.8%) included a description of a qualitative measure of acceptability, 157 (26.2%) reported a qualitative outcome related to acceptability, 109 (18.2%) included a description of how intervention attendance rates were captured, 199 (33.2%) reported intervention attendance, 162 (27%) included a description of how intervention compliance was measured, 187 (31.2%) reported intervention compliance, 23 (3.8%) provided information about the monetary cost of the intervention, 109 (18.2%) provided a description of how treatment fidelity was assessed, and 85 (14.2%) reported outcomes related to treatment fidelity.

### Reporting of feasibility indicators across time

Results from univariate logistic regression models for reporting feasibility indicators across time are communicated in Table 4. When compared to the Early Group (1982–2006), preliminary studies in the Late Group (2018–2020) tended to be more likely to report recruitment data (OR=1.60, 95%CI 1.03–2.49), and they were significantly more likely to report descriptions of quantitative measures (OR=3.05, 95%CI 1.97–4.72) and qualitative measures (OR=3.32, 95%CI 2.04–5.40) of acceptability, acceptability-related quantitative (OR=2.68, 95%CI 1.76–4.08) and qualitative (OR=2.32, 95%CI 1.48–3.65) outcomes, descriptions of compliance measures (OR=2.04, 95%CI 1.30–3.20) and compliance outcomes (OR=2.29, 95%CI 1.49–3.52), as well as descriptions of fidelity-related measures (OR=2.56, 95%CI 1.48–4.42) and fidelity outcomes (OR=2.13, 95%CI 1.21, 3.77). Late Group (2018–2020) studies were also significantly more likely to mention feasibility-related parameters in the purpose statement (OR=2.39, 95%CI 1.58–3.61) and to have cited a guideline or framework related to the reporting of preliminary studies (OR=28.7, 95%CI 6.87, 120.3). Marginal predicted probabilities for the reporting of feasibility indicators are communicated in Table 5. For each successive time period, the probability of reporting feasibility indicators significantly increased for all indicators but attendance.

**Table 4:** Summary of univariate logistic regression analysis for reporting feasibility indicators in included studies across time (*N=*600)

Feasibility indicator	Middle Group (2011–2013)^a^			Late Group (2018–2020)^a^		
	*N=*200			*N*=200		
	Odds ratio	95% CI^b^	*p*-value	Odds ratio	95% CI^b^	*p*-value
Recruitment	1.18	0.77, 1.79	0.450	1.60	1.03, 2.49	0.036
Retention^c^	1.51	0.25, 9.1	0.655	-	-	
Acceptability						
Description of quantitative measure	1.49	0.95, 2.35	0.085	**3.05**	**1.97, 4.72**	**<0.001**
Quantitative outcome	1.53	0.99, 2.35	0.051	**2.68**	**1.76, 4.08**	**<0.001**
Description of qualitative measure	1.57	0.93, 2.63	0.090	**3.32**	**2.04, 5.40**	**<0.001**
Qualitative outcome	1.23	0.76, 1.99	0.393	**2.32**	**1.48, 3.65**	**<0.001**
Attendance						
Description of measure	1.16	0.68, 1.96	0.590	1.49	0.89, 2.49	0.124
Outcome	1.14	0.76, 1.73	0.527	0.96	0.63, 1.45	0.830
Compliance						
Description of measure	1.36	0.85, 2.17	0.194	**2.04**	**1.30, 3.20**	**0.002**
Outcome	1.20	0.77, 1.88	0.424	**2.29**	**1.49, 3.52**	**<0.001**
Cost	0.62	0.19,1.91	0.261	1.26	0.49,3.27	0.193
Treatment fidelity						
Description of measure	**1.96**	**1.11, 3.45**	**0.019**	**2.56**	**1.48, 4.42**	**0.001**
Outcome	1.16	0.62, 2.16	0.635	2.13	1.21, 3.77	0.009
Mentioned feasibility-related parameters in purpose statement	1.38	0.93, 2.05	0.109	**2.38**	**1.58, 3.61**	**<0.001**
Cited a guideline/framework for the reporting of preliminary studies	**5.76**	**1.26, 26.3**	**0.024**	**28.74**	**6.87, 120.3**	**<0.001**

*Note*: ^a^Early Group (1982–2006, *N*=200) is the referent group

^b^95% CI stands for 95% confidence interval

^c^Late Group (2018–2020) retention variable had no observations coded as 0. Bold denotes significance at the *p* < 0.005 level

**Table 5:** Predicted probabilities for reporting feasibility indicators across time

Feasibility indicator	Early Group (1982–2006)		Middle Group (2011–2013)		Late Group (2018–2020)	
	*N*=200		*N*=200		*N*=200	
	Margin^a^	95% CI^b^	Margin^a^	95% CI^b^	Margin^a^	95% CI^b^
Recruitment	0.67	0.60–0.74	0.70	0.64–0.77	0.76	0.71–0.82
Retention^c^	0.98	0.97–1.00	0.99	0.98–1.00	-	-
Acceptability						
Description of quantitative measure	0.21	0.16–0.27	0.29	0.23–0.35	0.45	0.39–0.52
Quantitative outcome	0.26	0.19–0.32	0.35	0.28–0.42	0.49	0.42–0.55
Description of qualitative measure	0.15	0.09–0.19	0.21	0.15–0.27	0.36	0.29–0.43
Qualitative outcome	0.19	0.14–0.25	0.23	0.17–0.29	0.36	0.29–0.43
Attendance						
Description of measure	0.15	0.10–0.21	0.18	0.12–0.23	0.22	0.16–0.27
Outcome	0.33	0.26–0.39	0.36	0.29–0.42	0.32	0.25–0.38
Compliance						
Description of measure	0.21	0.15–0.26	0.26	0.19–0.32	0.35	0.28–0.41
Outcome	0.24	0.18–0.29	0.28	0.21–0.34	0.42	0.35–0.49
Cost	0.17	0.12–0.23	0.19	0.15–0.25	0.23	0.20–0.31
Treatment fidelity						
Description of measure	0.11	0.07–0.15	0.19	0.14–0.25	0.24	0.18–0.29
Outcome	0.11	0.06–0.15	0.12	0.07–0.17	0.20	0.14–0.26
Mentioned feasibility-related parameters in purpose statement	0.50	0.43–0.57	0.58	0.51–0.65	0.71	0.64–0.77
Cited a guideline/framework for the reporting of preliminary studies	0.01	−0.01–0.02	0.05	0.02–0.09	0.23	0.17–0.28

*Note*: ^a^The margin column communicates marginal predicted probabilities calculated from logistic regression models

^b^95% CI stands for 95% confidence interval

^c^Late Group (2018–2020) retention variable had no observations coded as 0

### Reporting of feasibility indicators and purpose statements, framework usage, and funding

Results from multivariate Poisson regression models for the number of reported feasibility indicators are presented in Table 6. Reporting funding, mentioning feasibility-related indicators in the purpose statement, and citing guidelines/frameworks for the reporting of preliminary studies all significantly and positively associated with the number of feasibility indicators reported. These relationships held after controlling for time period.

**Table 6:** Parameter estimates from Poisson regression models predicting the number of feasibility indicators reported in pilot and feasibility studies

Predictor	Model 1			Model 2		
	b-Coefficient	95% C.I.	*p-*value	b-Coefficient	95% C.I.	*p-*value
Funding reported	0.12	0.02–0.22	0.017	0.10	0.01–0.21	0.042
Mentioning feasibility-related indicators in purpose statement	**0.31**	**0.21**–**0.41**	**<0.001**	**0.29**	**0.19**–**0.39**	**<0.001**
Citing guidelines and/or frameworks for the reporting of preliminary studies.	**0.22**	**0.08**–**0.36**	**0.002**	0.17	0.03–0.32	0.019
Time period						
Middle Group (2011–2013)	-	-	-	0.04	−0.08–0.16	0.498
Late Group (2018–2020)	-	-	-	0.12	0.01–0.24	0.047

*Note*: 95% C.I. stands for 95% confidence interval; Model 2 = Model 1 + time periods (Early Group as referent). Bold denotes significance at the *p* < 0.005 level

## Discussion

This was a historical scoping review of the reporting of feasibility indicators in a large sample of obesity-related behavioral pilot/feasibility studies published between 1982 and 2020. We describe trends in the amount and type of feasibility indicators reported in studies across three time periods evaluating 200 studies from each period: 1982–2006, 2011–2013, and 2018–2020. Improvements over time were found for the reporting of most feasibility indicators; however, the rates of reporting remain modest, even in the latest group of studies published from 2018 to 2020. The majority of obesity-related behavioral pilot studies reported three or fewer feasibility outcomes, the most common being recruitment and retention, while almost all studies conducted/reported statistical analyses related to preliminary efficacy.

The primary finding from this study was the suboptimal rate of reporting key feasibility indicators within obesity-related behavioral pilot/feasibility studies. While *trial-related* feasibility was reported in the majority of studies (recruitment and/or retention), key *intervention-related* feasibility indicators, including participant acceptability, adherence, attendance, and intervention fidelity, were not widely reported. These results are supported by several reviews of pilot/feasibility studies conducted in other domains [34, 35], which all found a lack of *trial-* and *intervention-related* feasibility indicator reporting as well. While recruitment and retention are important *trial-related* feasibility indicators to capture, *intervention-related* feasibility indicators are important to assess during the preliminary phases of implementation. For example, participants’ perceptions of programs (acceptability) are associated with rates of attrition [36], intervention attendance is positively associate with obesity-related health outcomes [37, 38], and implementation fidelity during a pilot/feasibility study is associated with obesity-related main outcomes in scaled-up trials [39–42] and is shown to moderate the association between participant acceptability and behavioral outcomes [43].

The lack of reporting feasibility indicators coupled with the high rate of statistical testing for preliminary efficacy is concerning as well, although this does seem to be common across domains. For example, in a review of nursing intervention-feasibility literature, Mailhot et al. [34] found that almost half of the included feasibility studies focused exclusively on testing effectiveness. While preliminary efficacy can be reported in pilot/feasibility studies, results should be interpreted with caution, and outcomes related to feasibility should take priority.

Results from our study suggest that this is largely not the case in the behavioral sciences and reasons why remain unclear. It could be that intervention funders are invested in the outcome data. In other words, those agencies which fund preliminary studies might want some evidence that the intervention will have beneficial impact (regardless of its precision) before they continue to invest considerable time and money in a large, definitive trial. Several published guidelines, checklists, frameworks, and recommendations for pilot/feasibility studies exist [2, 5, 7, 10, 11, 44–46], many of which argue against the use of and focus on statistical testing for preliminary efficacy. However, pilot/feasibility studies have only just recently garnered attention from larger agencies. For example, the CONSORT extension to randomized pilot and feasibility trials [10] was published in 2016 and the majority of other literature that is used to guide pilot/feasibility studies has been published within the last decade as well. For pilot/feasibility studies included in this review, the most commonly cited guidelines/frameworks included the Medical Research Council guidance [1], the CONSORT extension for pilot and feasibility studies [10], and the RE-AIM framework [33]. Other guidelines used less often included Bowen et al. [12], Thabane et al. [5], and Arain et al. [28] Researchers conducting obesity-related preliminary studies today are encouraged to use the available literature in an effort to design high-quality preliminary interventions that can provide rich data to support the successful scaling up to a larger trial.

While our review does highlight some concerns for obesity-related behavioral pilot/feasibility studies, there were also some encouraging findings. We found studies conducted between 2018 and 2020 had higher odds of reporting most feasibility indicators when compared to studies published between 1982 and 2006. While reporting was still only modest in the later studies, results do show that improvements are occurring among behavioral pilot/feasibility studies. This may coincide with recent initiatives that have been undertaken in the field, including the publishing of several frameworks, guidelines, and recommendations related to pilot/feasibility studies [2, 5, 7, 10–12, 18, 45, 46]. Our results demonstrate that the reporting of feasibility indicators positively associated with citing a guideline/framework for the reporting of preliminary studies. Researchers conducting pilot/feasibility studies should utilize these guidelines/frameworks to inform the design, conduct, and reporting of their preliminary work, as our results support the idea that these guidelines/frameworks can improve the completeness of reporting in pilot/feasibility studies. We also found that the reporting of feasibility indicators positively associated with mentioning feasibility-related outcomes in the purpose statement of the published pilot/feasibility study. This may demonstrate the importance of stating clear objectives. Alternatively, it may also suggest that authors of these papers were generally more sensitized to the need to be explicit about aspects of feasibility.

The reporting of feasibility indicators also significantly and positively associated with a study being supported by funding of any kind. It is well established that pilot/feasibility studies play an essential role in the development of larger-scale trials and virtually all funding agencies require evidence gathered from these preliminary studies to support the justification for scaling up to a larger trial. This highlights the importance of funding structures that are designed to support the conduct of pilot/feasibility studies specifically. Recent initiatives like the NIH Planning Grant program (R34) [47], the CIHR Health Research Training Platform (HRTP) Pilot Funding Opportunity [48], and the NIHR Research for Patient Benefit (RfPB) program [49] represent important steps forward in the field of pilot/feasibility research.

## Strengths and limitations

A strength of this review is the inclusion of a large sample (*N=*600) of obesity-related pilot/feasibility studies published across four decades. Even though this was a scoping review and not every study published between 1982 and 2020 was included, we did not limit the inclusion of studies based on location, design, or health behavior topic, as long as the intervention contained at least one component related to obesogenic behaviors. As such, results can be generalized to a larger audience of health behavior researchers. There were also limitations to this review. First, we only considered health behavior interventions related to obesity for inclusion. While results may generalize to pilot/feasibility studies in the realm of health behavior, they cannot apply to non-behavioral preliminary studies including mechanistic, pharmacological, or rehabilitation interventions. Another limitation is that studies in the Early Group span a much greater length of time (1982–2006) compared to studies in the Middle (2011–2013) and Late (2018–2020) Groups and each year is not equally represented. Because of this grouping structure, comparisons between each time period, especially between the Early Group and Late Group, should be interpreted with caution. This was a function of the limited number of pilot/feasibility studies published in earlier years compared to later years. Also, due to the multi-stage randomization procedure used to screen studies for this scoping review, there were 9492 citations which were never screened. It must also be noted that some feasibility indicators may not have been relevant to collect for certain intervention designs. For example, attendance would most likely not have been an applicable feasibility indicator for an mHealth intervention, but participant compliance may have been a feasibility indicator of interest. In other words, depending on intervention design and the specific components of each pilot/feasibility study, it would be impossible or irrelevant for some studies to collect 100% of the feasibility indicators for which we coded. Furthermore, some of the feasibility indicators are difficult to code. We used both text mining and manual approaches to maximize accuracy in capturing this information, but some items may have been erroneous. Finally, reporting of a study is not identical to the conduct of a study. Not reporting of some aspect does not mean that the study authors had not taken it into consideration.

## Conclusions

The reporting of feasibility indicators within obesity-related behavioral pilot/feasibility studies has improved over time, but key aspects *of intervention-related* feasibility are still not reported in the majority of studies. Aspects of *intervention-related* feasibility, including fidelity, play a key role in the development of larger-scale trials, alongside the widely reported *trial-related* feasibility indicators of recruitment and retention. Given the importance of behavioral intervention pilot/feasibility studies in the translational science spectrum, there is a need for improving the reporting of feasibility indicators. Researchers who plan to conduct a pilot/feasibility trial with the intent to scale up to a future larger trial are encouraged to use the available literature on the design, conduct, and reporting of preliminary studies to improve design and maximize the potential success of the larger-scale trial.

## Supplementary Information

**Additional file 1: Supplementary File 1.** List of Included Studies.

**Additional file 2: Supplementary File 2.** PRISMA-ScR Checklist.

**Additional file 3: Supplementary File 3.** PubMed Example Search Strategy.

## Data Availability

The datasets, code, and other study materials used and analyzed are freely available at https://osf.io/5m8zr/.

## Abbreviations

CIHR: Canadian Institutes of Health Research
CONSORT: Consolidated Reporting of Trials
MeSH: Medical Subject Headings
MRC: Medical Research Council
NHMRC: National Health and Medical Research Council
NIH: National Institutes of Health
NIHR: National Institutes of Health Research
PRECIS: Pragmatic Explanatory Continuum Indicator Summary
PRISMA: Preferred Reporting Items for Systematic Reviews and Meta-Analyses
PRISMA-ScR: Preferred Reporting Items of Systematic Reviews and Meta-Analyses for scoping reviews
QUERI: Quality Enhancement Research Initiation
RCT: Randomized controlled trial
TIDieR: Template for Intervention Description and Replication
