# Supplementary material for: Feasibility indicators in obesity-related behavioral intervention preliminary studies: a historical scoping review
Source: Pilot Feasibility Stud. 2023 Mar 22;9:46. doi: 10.1186/s40814-023-01270-w (PMC10032007; doi:10.1186/s40814-023-01270-w)
Supplement: Supplementary file 1 — Additional file 1: Supplementary File 1. List of Included Studies. [file 40814_2023_1270_MOESM1_ESM.docx]

1. Aas AM, Bergstad I, Thorsbyt PM, Johannesen Ø, Solberg M, Birkelandt KI. An intensified lifestyle intervention programme may be superior to insulin treatment in poorly controlled Type 2 diabetic patients on oral hypoglycaemic agents: Results of a feasibility study. *Diabetic Medicine.* 2005;22(3):316-322.

2. Abu-Saad K, Murad H, Barid R, et al. Development and Efficacy of an Electronic, Culturally Adapted Lifestyle Counseling Tool for Improving Diabetes-Related Dietary Knowledge: Randomized Controlled Trial Among Ethnic Minority Adults With Type 2 Diabetes Mellitus. *J Med Internet Res.* 2019;21(10):e13674.

3. Ackerman E, Falsetti SA, Lewis P, Hawkins AO, Heinschel JA. Motivational interviewing: a behavioral counseling intervention for the family medicine provider. *Fam Med.* 2011;43(8):582-585.

4. Addison CC, Jenkins BW, White MS, Young L. Implementation of a cardiovascular disease prevention program among school-aged children: a pilot study. *Int J Environ Res Public Health.* 2006;3(3):274-277.

5. Ahmed S, Dupuis V, Tyron M, et al. Intended and Unintended Consequences of a Community-Based Fresh Fruit and Vegetable Dietary Intervention on the Flathead Reservation of the Confederated Salish and Kootenai Tribes. *Front Public Health.* 2020;8:331.

6. Akers J, Cornett RA, Savla J, Davy BM. Daily self-monitoring of body weight, step count, fruit/vegetable intake and water consumption: A feasible and effective long-term weight loss maintenance approach. *Obesity.* 2011;19:S100.

7. Al Khatib HK, Hall WL, Creedon A, et al. Sleep extension is a feasible lifestyle intervention in free-living adults who are habitually short sleepers: a potential strategy for decreasing intake of free sugars? A randomized controlled pilot study. *Am J Clin Nutr.* 2018;107(1):43-53.

8. Alert MD, Rastegar S, Foret M, et al. The effectiveness of a comprehensive mind body weight loss intervention for overweight and obese adults: a pilot study. *Complement Ther Med.* 2013;21(4):286-293.

9. Allison PJ, Edgar L, Nicolau B, Archer J, Black M, Hier M. Results of a feasibility study for a psycho-educational intervention in head and neck cancer. *Psychooncology.* 2004;13(7):482-485.

10. Almas A, Islam M, Jafar TH. School-based physical activity programme in preadolescent girls (9-11 years): a feasibility trial in Karachi, Pakistan. *Arch Dis Child.* 2013;98(7):515-519.

11. Ames GE, Perri MG, Fox LD, et al. Changing weight-loss expectations: a randomized pilot study. *Eat Behav.* 2005;6(3):259-269.

12. Andrews M, Sawyer C, Frerichs L, et al. Feasibility of a Clinic-Community Partnership to Treat Childhood Obesity. *Clinical Pediatrics.* 2018;57(7):783-791.

13. Aoun S, Osseiran-Moisson R, Shahid S, Howat P, M OC. Telephone lifestyle coaching: is it feasible as a behavioural change intervention for men? *J Health Psychol.* 2012;17(2):227-236.

14. Arauz Boudreau AD, Kurowski DS, Gonzalez WI, Dimond MA, Oreskovic NM. Latino families, primary care, and childhood obesity: a randomized controlled trial. *Am J Prev Med.* 2013;44(3 Suppl 3):S247-257.

15. Archie S, Wilson JH, Osborne S, Hobbs H, McNiven J. Pilot study: access to fitness facility and exercise levels in olanzapine-treated patients. *Can J Psychiatry.* 2003;48(9):628-632.

16. Armstrong K, Edwards H. The effects of exercise and social support on mothers reporting depressive symptoms: a pilot randomized controlled trial. *Int J Ment Health Nurs.* 2003;12(2):130-138.

17. Arrebola E, Gómez-Candela C, Fernández-Fernández C, Loria V, Muñoz-Pérez E, Bermejo LM. Evaluation of a lifestyle modification program for treatment of overweight and nonmorbid obesity in primary healthcare and its influence on health-related quality of life. *Nutr Clin Pract.* 2011;26(3):316-321.

18. Atlantis E, Chow CM, Kirby A, Singh MAF. Worksite intervention effects on physical health: a randomized controlled trial. *Health Promotion International.* 2006;21(3):191-200.

19. Ball K, Salmon J, Leslie E, Owen N, King AC. Piloting the feasibility and effectiveness of print- and telephone-mediated interventions for promoting the adoption of physical activity in Australian adults. *J Sci Med Sport.* 2005;8(2):134-142.

20. Banks-Wallace J, Conn V. Changes in steps per day over the course of a pilot walking intervention. *Abnf j.* 2005;16(2):28-32.

21. Baranowski T, Baranowski JC, Cullen KW, et al. The Fun, Food, and Fitness Project (FFFP): the Baylor GEMS pilot study. *Ethn Dis.* 2003;13(1 Suppl 1):S30-39.

22. Barnason S, Zimmerman L, Nieveen J, Schmaderer M, Carranza B, Reilly S. Impact of a home communication intervention for coronary artery bypass graft patients with ischemic heart failure on self-efficacy, coronary disease risk factor modification, and functioning. *Heart Lung.* 2003;32(3):147-158.

23. Barone Gibbs B, Hergenroeder AL, Perdomo SJ, Kowalsky RJ, Delitto A, Jakicic JM. Reducing sedentary behaviour to decrease chronic low back pain: the stand back randomised trial. *Occup Environ Med.* 2018;75(5):321-327.

24. Barr-Anderson DJ, Laska MN, Veblen-Mortenson S, Farbakhsh K, Dudovitz B, Story M. A school-based, peer leadership physical activity intervention for 6th graders: feasibility and results of a pilot study. *J Phys Act Health.* 2012;9(4):492-499.

25. Baruth M, Schlaff RA, Deere S, et al. The Feasibility and Efficacy of a Behavioral Intervention to Promote Appropriate Gestational Weight Gain. *Maternal and child health journal.* 2019;23(12):1604-1612.

26. Baschung Pfister P, Niedermann K, Sidelnikov E, Bischoff-Ferrari HA. Active over 45: a step-up jogging programme for inactive female hospital staff members aged 45+. *Eur J Public Health.* 2013;23(5):817-822.

27. Basen-Engquist K, Taylor CLC, Rosenblum C, et al. Randomized pilot test of a lifestyle physical activity intervention for breast cancer survivors. *Patient Education and Counseling.* 2006;64(1-3):225-234.

28. Batsis JA, McClure AC, Weintraub AB, et al. Feasibility and acceptability of a rural, pragmatic, telemedicine-delivered healthy lifestyle programme. *Obes Sci Pract.* 2019;5(6):521-530.

29. Bauer JD, Capra S. Nutrition intervention improves outcomes in patients with cancer cachexia receiving chemotherapy - A pilot study. *Supportive Care in Cancer.* 2005;13(4):270-274.

30. Baum EE, Jarjoura D, Polen AE, Faur D, Rutecki G. Effectiveness of a group exercise program in a long-term care facility: a randomized pilot trial. *J Am Med Dir Assoc.* 2003;4(2):74-80.

31. Bean MK, Ingersoll KS, Powell P, et al. Impact of motivational interviewing on outcomes of an adolescent obesity treatment: results from the MI Values randomized controlled pilot trial. *Clin Obes.* 2018;8(5):323-326.

32. Beebe LH, Tian L, Morris N, Goodwin A, Allen SS, Kuldau J. Effects of exercise on mental and physical health parameters of persons with schizophrenia. *Issues Ment Health Nurs.* 2005;26(6):661-676.

33. Beech BM, Klesges RC, Kumanyika SK, et al. Child- and parent-targeted interventions: the Memphis GEMS pilot study. *Ethn Dis.* 2003;13(1 Suppl 1):S40-53.

34. Bei B, Byrne ML, Ivens C, et al. Pilot study of a mindfulness-based, multi-component, in-school group sleep intervention in adolescent girls. *Early Interv Psychiatry.* 2013;7(2):213-220.

35. Bell BM, Martinez L, Gotsis M, et al. Virtual Sprouts: A Virtual Gardening Pilot Intervention Increases Self-Efficacy to Cook and Eat Fruits and Vegetables in Minority Youth. *Games Health J.* 2018;7(2):127-135.

36. Bellamy J, Broderick C, Hardy LL, et al. Feasibility of a school-based exercise intervention for children with intellectual disability to reduce cardio-metabolic risk. *J Intellect Disabil Res.* 2020;64(1):7-17.

37. Benloucif S, Orbeta L, Ortiz R, et al. Morning or evening activity improves neuropsychological performance and subjective sleep quality in older adults. *Sleep.* 2004;27(8):1542-1551.

38. Benyshek DC, Chino M, Dodge-Francis C, Begay TO, Jin H, Giordano C. Prevention of type 2 diabetes in urban American Indian/Alaskan Native communities: The Life in BALANCE pilot study. *J Diabetes Mellitus.* 2013;3(4):184-191.

39. Berger AM, VonEssen S, Khun BR, et al. Feasibilty of a sleep intervention during adjuvant breast cancer chemotherapy. *Oncol Nurs Forum.* 2002;29(10):1431-1441.

40. Berkley-Patton J, Bowe Thompson C, Bauer AG, et al. A Multilevel Diabetes and CVD Risk Reduction Intervention in African American Churches: Project Faith Influencing Transformation (FIT) Feasibility and Outcomes. *J Racial Ethn Health Disparities.* 2020;7(6):1160-1171.

41. Bick D, Taylor C, Bhavnani V, et al. Lifestyle information and commercial weight management groups to support maternal postnatal weight management and positive lifestyle behaviour: the SWAN feasibility randomised controlled trial. *Bjog.* 2020;127(5):636-645.

42. Biffi A, Fernando F, Adami PE, et al. Ferrari Corporate Wellness Program: Results of a Pilot Analysis and the "Drag" Impact in the Workplace. *High Blood Press Cardiovasc Prev.* 2018;25(3):261-266.

43. Blair CK, Madan-Swain A, Locher JL, et al. Harvest for health gardening intervention feasibility study in cancer survivors. *Acta Oncol.* 2013;52(6):1110-1118.

44. Block G, Block T, Wakimoto P, Block CH. Demonstration of an E-mailed worksite nutrition intervention program. *Prev Chronic Dis.* 2004;1(4):A06.

45. Blocker EM, Fry AC, Luebbers PE, et al. Promoting Alzheimer's Risk-Reduction through Community-Based Lifestyle Education and Exercise in Rural America: A Pilot Intervention. *Kans J Med.* 2020;13:179-185.

46. Blonstein AC, Yank V, Stafford RS, Wilson SR, Rosas LG, Ma J. Translating an evidence-based lifestyle intervention program into primary care: lessons learned. *Health Promot Pract.* 2013;14(4):491-497.

47. Bloom I, Welch L, Vassilev I, et al. Findings from an exploration of a social network intervention to promote diet quality and health behaviours in older adults with COPD: a feasibility study. *Pilot Feasibility Stud.* 2020;6:15.

48. Bogart LM, Elliott MN, Uyeda K, Hawes-Dawson J, Klein DJ, Schuster MA. Preliminary healthy eating outcomes of SNaX, a pilot community-based intervention for adolescents. *J Adolesc Health.* 2011;48(2):196-202.

49. Booth AO, Nowson CA, Huang N, Lombard C, Singleton KL. Evaluation of a brief pilot nutrition and exercise intervention for the prevention of weight gain in general practice patients. *Public Health Nutr.* 2006;9(8):1055-1061.

50. Branscum P, Sharma M, Wang LL, Wilson BR, Rojas-Guyler L. A true challenge for any superhero: an evaluation of a comic book obesity prevention program. *Fam Community Health.* 2013;36(1):63-76.

51. Brown B, Noonan C, Harris KJ, et al. Developing and piloting the Journey to Native Youth Health program in Northern Plains Indian communities. *Diabetes Educ.* 2013;39(1):109-118.

52. Brown SA, García AA, Winter M, Silva L, Brown A, Hanis CL. Integrating education, group support, and case management for diabetic Hispanics. *Ethn Dis.* 2011;21(1):20-26.

53. Brown SA, Hanis CL. A community-based, culturally sensitive education and group-support intervention for Mexican Americans with NIDDM: a pilot study of efficacy. *Diabetes Educ.* 1995;21(3):203-210.

54. Brown WJ, Lee C. Exercise and dietary modification with women of non-English speaking background: a pilot study with Polish-Australian women. *Int J Behav Med.* 1994;1(3):185-203.

55. Buchholz SW, Wilbur J, Miskovich L, Gerard P. An office-based health promotion intervention for overweight and obese uninsured adults: a feasibility study. *J Cardiovasc Nurs.* 2012;27(1):68-75.

56. Bullen K, Benton D. A pilot study to explore the challenges of changing children's food and health concepts. *Health Education Journal.* 2004;63(1):50-60.

57. Bünger J, Lanzerath I, Ruhnau P, et al. Company health care: Evaluation of concepts for reducing cardiovascular risks. *Arbeitsmedizin Sozialmedizin Umweltmedizin.* 2003;38(8):421-425.

58. Burnet DL, Plaut AJ, Wolf SA, et al. Reach-Out: A Family-Based Diabetes Prevention Program for African American Youth. *Journal of the National Medical Association.* 2011;103(3):269-277.

59. Busch V, De Leeuw RJJ, Schrijvers AJP. Results of a multibehavioral health-promoting school pilot intervention in a dutch secondary school. *Journal of Adolescent Health.* 2013;52(4):400-406.

60. Butryn ML, Forman E, Hoffman K, Shaw J, Juarascio A. A pilot study of acceptance and commitment therapy for promotion of physical activity. *J Phys Act Health.* 2011;8(4):516-522.

61. Buzzard IM, Asp EH, Chlebowski RT, et al. Diet intervention methods to reduce fat intake: Nutrient and food group composition of self-selected low-fat diets. *Journal of the American Dietetic Association.* 1990;90(1):42-50+53.

62. Caine-Bish N, Gordon KL. Calcium and KIDS: A Pilot Program Developed to Increase Calcium Intakes in Third- through Sixth-Grade Children. *Journal of Nutrition Education and Behavior.* 2006;38(3):199-200.

63. Campbell KL, Van Patten CL, Neil SE, et al. Feasibility of a lifestyle intervention on body weight and serum biomarkers in breast cancer survivors with overweight and obesity. *J Acad Nutr Diet.* 2012;112(4):559-567.

64. Campbell MK, Honess-Morreale L, Farrell D, Carbone E, Brasure M. A tailored multimedia nutrition education pilot program for low-income women receiving food assistance. *Health Educ Res.* 1999;14(2):257-267.

65. Carlin A, Murphy MH, Gallagher AM. Using the School Environment to Promote Walking amongst Adolescent Females: A Mixed-Method Study. *Children (Basel).* 2019;6(3).

66. Carpenter KM, Vickerman KA, Salmon EE, Javitz HS, Epel ES, Lovejoy JC. A Randomized Pilot Study of a Phone-Based Mindfulness and Weight Loss Program. *Behav Med.* 2019;45(4):271-281.

67. Carpenter RA, Finley C, Barlow CE. Pilot test of a behavioral skill building intervention to improve overall diet quality. *J Nutr Educ Behav.* 2004;36(1):20-24.

68. Carr LJ, Walaska KA, Marcus BH. Feasibility of a portable pedal exercise machine for reducing sedentary time in the workplace. *British journal of sports medicine.* 2012;46(6):430-435.

69. Carroll JK, Fiscella K, Epstein RM, et al. Physical activity counseling intervention at a federally qualified health center: improves autonomy-supportiveness, but not patients' perceived competence. *Patient Educ Couns.* 2013;92(3):432-436.

70. Carter MC, Burley VJ, Nykjaer C, Cade JE, Eysenbach G. Adherence to a Smartphone Application for Weight Loss Compared to Website and Paper Diary: Pilot Randomized Controlled Trial. *Journal of Medical Internet Research.* 2013;15(4):1-1.

71. Carter SE, Draijer R, Maxwell JD, et al. Using an e-Health Intervention to Reduce Prolonged Sitting in UK Office Workers: A Randomised Acceptability and Feasibility Study. *Int J Environ Res Public Health.* 2020;17(23).

72. Celano CM, Gianangelo TA, Millstein RA, et al. A positive psychology-motivational interviewing intervention for patients with type 2 diabetes: Proof-of-concept trial. *Int J Psychiatry Med.* 2019;54(2):97-114.

73. Cené CW, Haymore LB, Ellis D, et al. Implementation of the power to prevent diabetes prevention educational curriculum into rural African American communities: a feasibility study. *Diabetes Educ.* 2013;39(6):776-785.

74. Cerulli J, Zeolla MM. Impact and feasibility of a community pharmacy bone mineral density screening and education program. *J Am Pharm Assoc (2003).* 2004;44(2):161-167.

75. Chasan-Taber L, Silveira M, Marcus BH, Braun B, Stanek E, Markenson G. Feasibility and efficacy of a physical activity intervention among pregnant women: the behaviors affecting baby and you (B.A.B.Y.) study. *J Phys Act Health.* 2011;8 Suppl 2(0 2):S228-238.

76. Chen JC, Chang QX, Liang CC, et al. Potential benefits of environmental volunteering programs of the health of older adults: a pilot study. *Arch Gerontol Geriatr.* 2020;90:104113.

77. Chen JL, Weiss S, Heyman MB, Cooper B, Lustig RH. The efficacy of the web-based childhood obesity prevention program in Chinese American adolescents (Web ABC study). *J Adolesc Health.* 2011;49(2):148-154.

78. Chen S, Dzewaltowski DA, Rosenkranz RR, et al. Feasibility study of the SWITCH implementation process for enhancing school wellness. *BMC Public Health.* 2018;18(1):1119.

79. Chesham RA, Booth JN, Sweeney EL, et al. The Daily Mile makes primary school children more active, less sedentary and improves their fitness and body composition: a quasi-experimental pilot study. *BMC Med.* 2018;16(1):64.

80. Cheung NW, Smith BJ, van der Ploeg HP, Cinnadaio N, Bauman A. A pilot structured behavioural intervention trial to increase physical activity among women with recent gestational diabetes. *Diabetes Res Clin Pract.* 2011;92(1):e27-29.

81. Cheung YT, Lam TH, Chan CHH, et al. Brief handgrip and isometric exercise intervention for smoking cessation: A pilot randomized trial. *Addict Behav.* 2020;100:106119.

82. Chhugani KJ, Metri K, Babu N, Nagendra HR. Effects of Integrated Yoga Intervention on Psychopathologies and Sleep Quality Among Professional Caregivers of Older Adults With Alzheimer's Disease: A Controlled Pilot Study. *Adv Mind Body Med.* 2018;32(3):18-22.

83. Choi J, Fukuoka Y. Does having a buddy help women with young children increase physical activity? Lessons learned from a pilot study. *Women Health.* 2019;59(2):115-131.

84. Choi SE, Rush EB. Effect of a short-duration, culturally tailored, community-based diabetes self-management intervention for Korean immigrants: a pilot study. *Diabetes Educ.* 2012;38(3):377-385.

85. Choudhry S, McClinton-Powell L, Solomon M, et al. Power-up: a collaborative after-school program to prevent obesity in African American children. *Prog Community Health Partnersh.* 2011;5(4):363-373.

86. Chrisman SPD, Whitlock KB, Mendoza JA, et al. Pilot Randomized Controlled Trial of an Exercise Program Requiring Minimal In-person Visits for Youth With Persistent Sport-Related Concussion. *Front Neurol.* 2019;10:623.

87. Christaki E, Kokkinos A, Costarelli V, Alexopoulos EC, Chrousos GP, Darviri C. Stress management can facilitate weight loss in Greek overweight and obese women: a pilot study. *J Hum Nutr Diet.* 2013;26 Suppl 1:132-139.

88. Christison A, Khan HA. Exergaming for health: a community-based pediatric weight management program using active video gaming. *Clin Pediatr (Phila).* 2012;51(4):382-388.

89. Christopher KA, Morrow LL. Evaluating a community-based exercise program for women cancer survivors. *Appl Nurs Res.* 2004;17(2):100-108.

90. Chung LMY, Chung JWY, Chan APC. Building Healthy Eating Knowledge and Behavior: An Evaluation of Nutrition Education in a Skill Training Course for Construction Apprentices. *Int J Environ Res Public Health.* 2019;16(23).

91. Cioe PA, Guthrie KM, Freiberg MS, Williams DM, Kahler CW. Cardiovascular Risk Reduction in Persons Living With HIV: Treatment Development, Feasibility, and Preliminary Results. *J Assoc Nurses AIDS Care.* 2018;29(2):163-177.

92. Clark J, Craig L, McNeill G, Smith N, Norrie J, Devereux G. A novel dietary intervention to optimize vitamin E intake of pregnant women to 15 mg/Day. *Journal of the Academy of Nutrition and Dietetics.* 2012;112(2):297-301.

93. Coates RJ, Bowen DJ, Kristal AR, et al. The Women's Health Trial Feasibility Study in Minority Populations: changes in dietary intakes. *Am J Epidemiol.* 1999;149(12):1104-1112.

94. Cockcroft A, Gooch C, Ellinghouse C, Johnston M, Michie S. Evaluation of a programme of health measurements and advice among hospital staff. *Occup Med (Lond).* 1994;44(2):70-76.

95. Cody R, Lee C. Development and evaluation of a pilot program to promote exercise among mothers of preschool children. *Int J Behav Med.* 1999;6(1):13-29.

96. Cooley D, Pedersen S. A pilot study of increasing nonpurposeful movement breaks at work as a means of reducing prolonged sitting. *J Environ Public Health.* 2013;2013:128376.

97. Corepal R, Best P, O'Neill R, et al. A feasibility study of 'The StepSmart Challenge' to promote physical activity in adolescents. *Pilot Feasibility Stud.* 2019;5:132.

98. Corsino L, Rocha-Goldberg MP, Batch BC, Ortiz-Melo DI, Bosworth HB, Svetkey LP. The Latino Health Project: pilot testing a culturally adapted behavioral weight loss intervention in obese and overweight Latino adults. *Ethn Dis.* 2012;22(1):51-57.

99. Cortesi F, Giannotti F, Sebastiani T, Bruni O, Ottaviano S. Knowledge of sleep in Italian high school students: pilot-test of a school-based sleep educational program. *J Adolesc Health.* 2004;34(4):344-351.

100. Costa ABP, Rodrigues A, Martins LB, et al. Nutritional intervention may improve migraine severity: a pilot study. *Arq Neuropsiquiatr.* 2019;77(10):723-730.

101. Cotton B, Smith A, Hansen I, Davis C, Doyle A, Walsh A. Physician-directed primary care intervention to reduce risk factors for type 2 diabetes in high-risk youth. *Am J Med Sci.* 2006;332(3):108-111.

102. Cox TL, Krukowski R, Love SRJ, et al. Stress Management-Augmented Behavioral Weight Loss Intervention for African American Women: A Pilot, Randomized Controlled Trial. *Health Education and Behavior.* 2013;40(1):78-87.

103. Craigie AM, Barton KL, Macleod M, et al. A feasibility study of a personalised lifestyle programme (HealthForce) for individuals who have participated in cardiovascular risk screening. *Preventive Medicine.* 2011;52(5):387-389.

104. Cui Z, Shah S, Yan L, et al. Effect of a school-based peer education intervention on physical activity and sedentary behaviour in Chinese adolescents: a pilot study. *BMJ Open.* 2012;2(3).

105. Cupples ME, Cole JA, Hart ND, Heron N, McKinley MC, Tully MA. Shared decision-making (SHARE-D) for healthy behaviour change: a feasibility study in general practice. *BJGP Open.* 2018;2(2):bjgpopen18X101517.

106. Currie J, Collier D, Raedeke TD, Lutes LD, Kemble CD, Dubose KD. The effects of a low-dose physical activity intervention on physical activity and body mass index in severely obese adolescents. *International Journal of Adolescent Medicine and Health.* 2018;30(6).

107. Dağistan Akgöz A, Gözüm S. Effectiveness of a nurse-led physical activity intervention to decrease cardiovascular disease risk in middle-aged adults: A pilot randomized controlled study. *J Vasc Nurs.* 2020;38(3):140-148.

108. Daley AJ, Jolly K, Bensoussane H, et al. Feasibility and acceptability of a brief routine weight management intervention for postnatal women embedded within the national child immunisation programme in primary care: randomised controlled cluster feasibility trial. *Trials.* 2020;21(1):757.

109. Dämon S, Dietwch S, Widhalm K. PRESTO - Prevention Study of Obesity: A project to prevent obesity during childhood and adolescence. *Acta Paediatrica, International Journal of Paediatrics, Supplement.* 2005;94(448):47-48.

110. Das-Friebel A, Perkinson-Gloor N, Brand S, et al. A pilot cluster-randomised study to increase sleep duration by decreasing electronic media use at night and caffeine consumption in adolescents. *Sleep Med.* 2019;60:109-115.

111. Daumit GL, Dalcin AT, Jerome GJ, et al. A behavioral weight-loss intervention for persons with serious mental illness in psychiatric rehabilitation centers. *Int J Obes (Lond).* 2011;35(8):1114-1123.

112. David P, Buckworth J, Pennell ML, Katz ML, DeGraffinreid CR, Paskett ED. A walking intervention for postmenopausal women using mobile phones and Interactive Voice Response. *J Telemed Telecare.* 2012;18(1):20-25.

113. Davis AM, Gallagher K, Taylor M, et al. An in-home intervention to improve nutrition, physical activity, and knowledge among low-income teen mothers and their children: results from a pilot study. *J Dev Behav Pediatr.* 2013;34(8):609-615.

114. Davis AM, Sampilo M, Gallagher KS, Landrum Y, Malone B. Treating rural pediatric obesity through telemedicine: outcomes from a small randomized controlled trial. *J Pediatr Psychol.* 2013;38(9):932-943.

115. Davis JN, Ventura EE, Cook LT, Gyllenhammer LE, Gatto NM. LA Sprouts: a gardening, nutrition, and cooking intervention for Latino youth improves diet and reduces obesity. *J Am Diet Assoc.* 2011;111(8):1224-1230.

116. Davis SM, Going SB, Helitzer DL, et al. Pathways: a culturally appropriate obesity-prevention program for American Indian schoolchildren. *Am J Clin Nutr.* 1999;69(4 Suppl):796s-802s.

117. Davison KK, Edmunds LS, Wyker BA, Young LM, Sarfoh VS, Sekhobo JP. Feasibility of increasing childhood outdoor play and decreasing television viewing through a family-based intervention in WIC, New York State, 2007-2008. *Prev Chronic Dis.* 2011;8(3):A54.

118. Davison KK, Jurkowski JM, Li KG, Kranz S, Lawson HA. A childhood obesity intervention developed by families for families: results from a pilot study. *International Journal of Behavioral Nutrition and Physical Activity.* 2013;10:11.

119. de Blok BM, de Greef MH, ten Hacken NH, Sprenger SR, Postema K, Wempe JB. The effects of a lifestyle physical activity counseling program with feedback of a pedometer during pulmonary rehabilitation in patients with COPD: a pilot study. *Patient Educ Couns.* 2006;61(1):48-55.

120. De Jong NP, Debache I, Pan Z, et al. Breaking up Sedentary Time in Overweight/Obese Adults on Work Days and Non-Work Days: Results from a Feasibility Study. *Int J Environ Res Public Health.* 2018;15(11).

121. de la Haye K, Bell BM, Salvy S-J. The role of maternal social networks on the outcomes of a home-based childhood obesity prevention pilot intervention. *Journal of social structure : JOSS.* 2019;20(3):7-28.

122. Degroote L, Van Dyck D, De Bourdeaudhuij I, De Paepe A, Crombez G. Acceptability and feasibility of the mHealth intervention 'MyDayPlan' to increase physical activity in a general adult population. *BMC Public Health.* 2020;20(1):1032.

123. DeJoy DM, Padilla HM, Wilson MG, Vandenberg RJ, Davis MA. Worksite translation of the Diabetes Prevention Program: formative research and pilot study results from FUEL Your Life. *Health Promot Pract.* 2013;14(4):506-513.

124. Delamater AM, Pulgaron ER, Rarback S, et al. Web-based family intervention for overweight children: a pilot study. *Child Obes.* 2013;9(1):57-63.

125. deRosset L, Berry DC, Sanchez-Lugo L, et al. Mama sana … usted sana: lessons learned from a postpartum weight loss intervention for Hispanic women with infants six months or less. *Hisp Health Care Int.* 2013;11(2):78-86.

126. D'Haese S, Van Dyck D, De Bourdeaudhuij I, Cardon G. Effectiveness and feasibility of lowering playground density during recess to promote physical activity and decrease sedentary time at primary school. *BMC Public Health.* 2013;13.

127. Djuric Z, DiLaura NM, Jenkins I, et al. Combining weight-loss counseling with the weight watchers plan for obese breast cancer survivors. *Obes Res.* 2002;10(7):657-665.

128. Doering T, Harwell S, Fassler C, Burr K, Hewitt S, Trabue C. An interventional pilot study on obesity among low-income patients using a computer-based weight management module. *J Community Hosp Intern Med Perspect.* 2013;3(1).

129. Dopp RR, Mooney AJ, Armitage R, King C. Exercise for adolescents with depressive disorders: a feasibility study. *Depress Res Treat.* 2012;2012:257472.

130. Dowthwaite JN, Weiss DM, Thein-Nissenbaum J, Scerpella TA. A 2-yr, School-Based Resistance Exercise Pilot Program Increases Bone Accrual in Adolescent Girls. *Transl J Am Coll Sports Med.* 2019;4(11):74-83.

131. Doyle W, Crawford MA, Srivastava A, Costeloe KL. Interpregnancy nutrition intervention with mothers of low-birthweight babies living in an inner city area: A feasibility study. *Journal of Human Nutrition and Dietetics.* 1999;12(6):517-527.

132. Duarte N, Santos C, Hughes SL, Paúl C. Feasibility and impact of Fit & Strong! Program in Portuguese older adults with osteoarthritis: A pilot randomized controlled trial. *Geriatr Nurs.* 2020;41(6):804-811.

133. Dubbert PM, Cushman WC, Meydrech EF, Rowland AK, Maury P. Effects of dietary instruction and sodium excretion feedback in hypertension clinic patients. *Behavior Therapy.* 1995;26(4):721-732.

134. Duncan AD, Peters BS, Rivas C, Goff LM. Reducing risk of Type 2 diabetes in HIV: a mixed-methods investigation of the STOP-Diabetes diet and physical activity intervention. *Diabet Med.* 2020;37(10):1705-1714.

135. Duncan LR, Martinez JL, Rivers SE, et al. Healthy Eating for Life English as a second language curriculum: primary outcomes from a nutrition education intervention targeting cancer risk reduction. *J Health Psychol.* 2013;18(7):950-961.

136. Duncan S, McPhee JC, Schluter PJ, Zinn C, Smith R, Schofield G. Efficacy of a compulsory homework programme for increasing physical activity and healthy eating in children: the healthy homework pilot study. *Int J Behav Nutr Phys Act.* 2011;8:127.

137. Dupart G, Berry DC, D'Auria J, et al. A Nurse-Led and Teacher-Assisted Adolescent Healthy Weight Program to Improve Health Behaviors in the School Setting. *J Sch Nurs.* 2019;35(3):178-188.

138. DuVall C, Dinger MK, Taylor EL, Bemben D. Minimal-contact physical activity interventions in women: a pilot study. *Am J Health Behav.* 2004;28(3):280-286.

139. Eakin EG, Lawler SP, Winkler EA, Hayes SC. A randomized trial of a telephone-delivered exercise intervention for non-urban dwelling women newly diagnosed with breast cancer: exercise for health. *Ann Behav Med.* 2012;43(2):229-238.

140. Ebbeling CB, Feldman HA, Osganian SK, Chomitz VR, Ellenbogen SJ, Ludwig DS. Effects of decreasing sugar-sweetened beverage consumption on body weight in adolescents: a randomized, controlled pilot study. *Pediatrics.* 2006;117(3):673-680.

141. Ebbeling CB, Leidig MM, Sinclair KB, Seger-Shippee LG, Feldman HA, Ludwig DS. Effects of an ad libitum low-glycemic load diet on cardiovascular disease risk factors in obese young adults. *Am J Clin Nutr.* 2005;81(5):976-982.

142. Eiben G, Lissner L. Health Hunters - An intervention to prevent overweight and obesity in young high-risk women. *International Journal of Obesity.* 2006;30(4):691-696.

143. Eisenberg DM, Righter AC, Matthews B, Zhang W, Willett WC, Massa J. Feasibility Pilot Study of a Teaching Kitchen and Self-Care Curriculum in a Workplace Setting. *Am J Lifestyle Med.* 2019;13(3):319-330.

144. Elbel R, Aldana S, Bloswick D, Lyon JL. A pilot study evaluating a peer led and professional led physical activity intervention with blue-collar employees. *Work.* 2003;21(3):199-210.

145. Eliason MJ, Skinstad AH. Drug & alcohol intervention for older women: a pilot study. *J Gerontol Nurs.* 2001;27(12):18-24; quiz 40.

146. Elliot DL, Goldberg L, Duncan TE, et al. The PHLAME firefighters' study: feasibility and findings. *Am J Health Behav.* 2004;28(1):13-23.

147. Engel FA, Wagner MO, Schelhorn F, et al. Classroom-Based Micro-Sessions of Functional High-Intensity Circuit Training Enhances Functional Strength but Not Cardiorespiratory Fitness in School Children-A Feasibility Study. *Front Public Health.* 2019;7:291.

148. Englert HS, Diehl HA, Greenlaw RL. Rationale and design of the Rockford CHIP, a community-based coronary risk reduction program: results of a pilot phase. *Preventive Medicine.* 2004;38(4):432-441.

149. Entwistle TR, Green AC, Fildes JE, Miura K. Adherence to Mediterranean and low-fat diets among heart and lung transplant recipients: a randomized feasibility study. *Nutr J.* 2018;17(1):22.

150. Eriksson J, Lindstrom J, Valle T, et al. Prevention of Type II diabetes in subjects with impaired glucose tolerance: the Diabetes Prevention Study (DPS) in Finland - Study design and 1-year interim report on the feasibility of the lifestyle intervention programme. *Diabetologia.* 1999;42(7):793-801.

151. Eriksson KF, Lindgärde F. Prevention of type 2 (non-insulin-dependent) diabetes mellitus by diet and physical exercise. The 6-year Malmö feasibility study. *Diabetologia.* 1991;34(12):891-898.

152. Essa AR, Browne EP, Punska EC, et al. Dietary Intervention to Increase Fruit and Vegetable Consumption in Breastfeeding Women: A Pilot Randomized Trial Measuring Inflammatory Markers in Breast Milk. *J Acad Nutr Diet.* 2018;118(12):2287-2295.

153. Estabrooks PA, Fox EH, Doerksen SE, Bradshaw MH, King AC. Participatory research to promote physical activity at congregate-meal sites. *J Aging Phys Act.* 2005;13(2):121-144.

154. Evans AE, Dave J, Tanner A, et al. Changing the home nutrition environment: effects of a nutrition and media literacy pilot intervention. *Fam Community Health.* 2006;29(1):43-54.

155. Evans EW, Bond DS, Pierre DF, Howie WC, Wing RR, Jelalian E. Promoting health and activity in the summer trial: Implementation and outcomes of a pilot study. *Prev Med Rep.* 2018;10:87-92.

156. Faghanipour S, Hajikazemi E, Nikpour S, Shariatpanahi SAS, Hosseini AF. Mobile phone short message service (SMS) for weight management in Iranian overweight and obese women: A pilot study. *International Journal of Telemedicine and Applications.* 2013.

157. Fahrenwald NL, Atwood JR, Walker SN, Johnson DR, Berg K. A randomized pilot test of "Moms on the Move": a physical activity intervention for WIC mothers. *Ann Behav Med.* 2004;27(2):82-90.

158. Faith MS, Berman N, Heo M, et al. Effects of contingent television on physical activity and television viewing in obese children. *Pediatrics.* 2001;107(5):1043-1048.

159. Fani M, Mostamand J, Fani M, Chitsaz N, Feizi A. The effect of aerobic exercises among women with mild and moderate irritable bowel syndrome: A pilot study. *J Bodyw Mov Ther.* 2019;23(1):161-165.

160. Farbo D, Maler LC, Rhea DJ. The Preliminary Effects of a Multi-Recess School Intervention: Using Accelerometers to Measure Physical Activity Patterns in Elementary Children. *Int J Environ Res Public Health.* 2020;17(23).

161. Farrell K, Wicks MN, Martin JC. Chronic disease self-management improved with enhanced self-efficacy. *Clin Nurs Res.* 2004;13(4):289-308.

162. Ferrara A, Hedderson MM, Albright CL, et al. A pregnancy and postpartum lifestyle intervention in women with gestational diabetes mellitus reduces diabetes risk factors: a feasibility randomized control trial. *Diabetes Care.* 2011;34(7):1519-1525.

163. Fields JZ, Walton KG, Schneider RH, et al. Effect of a multimodality natural medicine program on carotid atherosclerosis in older subjects: a pilot trial of Maharishi Vedic Medicine. *Am J Cardiol.* 2002;89(8):952-958.

164. Fine A, Ward M, Burr M, Tudor-Smith C, Kingdon A. Health promotion in small workplaces - A feasibility study. *Health Education Journal.* 2004;63(4):334-346.

165. Fisher MC, Villegas E, Sutter C, Musaad SM, Koester B, Fiese BH. Sprouts Growing Healthy Habits: Curriculum Development and Pilot Study. *Front Public Health.* 2019;7:65.

166. Fitzgibbon ML, Stolley MR, Ganschow P, et al. Results of a faith-based weight loss intervention for black women. *J Natl Med Assoc.* 2005;97(10):1393-1402.

167. Fitzgibbon ML, Stolley MR, Schiffer L, et al. Family-based hip-hop to health: outcome results. *Obesity (Silver Spring).* 2013;21(2):274-283.

168. Fitzgibbon ML, Stolley MR, Schiffer L, Sanchez-Johnsen LAP, Wells AM, Dyer A. A combined breast health/weight loss intervention for Black women. *Preventive Medicine.* 2005;40(4):373-383.

169. Flannery K, Resnick B, Galik E, Lipscomb J, McPhaul K, Shaughnessy M. The Worksite Heart Health Improvement Project (WHHIP): feasibility and efficacy. *Public Health Nurs.* 2012;29(5):455-466.

170. Fleary S, Heffer RW, McKyer EL, Taylor A. A parent-focused pilot intervention to increase parent health literacy and healthy lifestyle choices for young children and families. *ISRN Family Med.* 2013;2013:619389.

171. Flores R. Dance for health: improving fitness in African American and Hispanic adolescents. *Public Health Rep.* 1995;110(2):189-193.

172. Ford BS, McDonald TE, Owens AS, Robinson TN. Primary care interventions to reduce television viewing in African-American children. *Am J Prev Med.* 2002;22(2):106-109.

173. Foster K, Stoeckle J, Silverio A, et al. Attitudes Surrounding a Community-Based Fitness Intervention at an Urban FQHC. *Fam Med.* 2019;51(7):598-602.

174. Fraticelli F, Nicola MD, Vitacolonna E. A nutritional web-based approach in obesity and diabetes before and during COVID-19 lockdown. *J Telemed Telecare.* 2020:1357633x20966933.

175. Frediani JK, Bienvenida AF, Li J, Higgins MK, Lobelo F. Physical fitness and activity changes after a 24-week soccer-based adaptation of the U.S diabetes prevention program intervention in Hispanic men. *Prog Cardiovasc Dis.* 2020;63(6):775-785.

176. Friel S, Kelleher C, Campbell P, Nolan G. Evaluation of the Nutrition Education at Primary School (NEAPS) programme. *Public Health Nutr.* 1999;2(4):549-555.

177. Fritz H, Tarraf W, Brody A, Levy P. Feasibility of a behavioral automaticity intervention among African Americans at risk for metabolic syndrome. *BMC Public Health.* 2019;19(1):413.

178. Fukuda H, Muto T, Kawamori R. Evaluation of a diabetes patient education program consisting of a three-day hospitalization and a six-month follow-up by telephone counseling for mild type 2 diabetes and IGT. *Environ Health Prev Med.* 1999;4(3):122-129.

179. Fukuoka Y, Vittinghoff E, Hooper J. A weight loss intervention using a commercial mobile application in latino americans-adelgaza trial. *Circulation.* 2018;137.

180. Garcia DO, Valdez LA, Aceves B, et al. A Gender- and Culturally Sensitive Weight Loss Intervention for Hispanic Men: Results From the Animo Pilot Randomized Controlled Trial. *Health Educ Behav.* 2019;46(5):763-772.

181. Gardiner PA, Eakin EG, Healy GN, Owen N. Feasibility of reducing older adults' sedentary time. *Am J Prev Med.* 2011;41(2):174-177.

182. Geaney F, Harrington J, Fitzgerald A, Perry I. The impact of a workplace catering initiative on dietary intakes of salt and other nutrients: a pilot study. *Public Health Nutr.* 2011;14(8):1345-1349.

183. Gentile N, Kaufman TK, Maxson J, et al. The Effectiveness of a Family-Centered Childhood Obesity Intervention at the YMCA: A Pilot Study. *J Community Med Health Educ.* 2018;8(1).

184. Gibson CA, Gupta A, Greene JL, Lee J, Mount RR, Sullivan DK. Feasibility and acceptability of a televideo physical activity and nutrition program for recent kidney transplant recipients. *Pilot Feasibility Stud.* 2020;6:126.

185. Gifford AL, Laurent DD, Gonzales VM, Chesney MA, Lorig KR. Pilot randomized trial of education to improve self-management skills of men with symptomatic HIV/AIDS. *J Acquir Immune Defic Syndr Hum Retrovirol.* 1998;18(2):136-144.

186. Gleeson-Kreig JM. Self-monitoring of physical activity: effects on self-efficacy and behavior in people with type 2 diabetes. *Diabetes Educ.* 2006;32(1):69-77.

187. Goldhaber-Fiebert JD, Goldhaber-Fiebert SN, Tristán ML, Nathan DM. Randomized controlled community-based nutrition and exercise intervention improves glycemia and cardiovascular risk factors in type 2 diabetic patients in rural Costa Rica. *Diabetes Care.* 2003;26(1):24-29.

188. Goodrich DE, Buis LR, Janney AW, et al. Integrating an internet-mediated walking program into family medicine clinical practice: a pilot feasibility study. *BMC Med Inform Decis Mak.* 2011;11:47.

189. Gordon MM, Thomson EA, Madhok R, Capell HA. Can intervention modify adverse lifestyle variables in a rheumatoid population? Results of a pilot study. *Ann Rheum Dis.* 2002;61(1):66-69.

190. Gorin A, Raynor H, Chula-Maguire K, Wing R. Decreasing household television time: A pilot study of a combined behavioral and environmental intervention. *Behavioral Interventions.* 2006;21(4):273-280.

191. Gotsis M, Wang H, Spruijt-Metz D, Jordan-Marsh M, Valente TW. Wellness partners: design and evaluation of a web-based physical activity diary with social gaming features for adults. *JMIR Res Protoc.* 2013;2(1):e10.

192. Gray CM, Hunt K, Mutrie N, Anderson AS, Treweek S, Wyke S. Weight management for overweight and obese men delivered through professional football clubs: a pilot randomized trial. *Int J Behav Nutr Phys Act.* 2013;10:121.

193. Gray SM, Chen P, Fleig L, et al. Can a Lifestyle Intervention Increase Active Transportation in Women Aged 55-70 years? Secondary Outcomes From a Pilot Randomized Controlled Trial. *J Phys Act Health.* 2018;15(6):411-416.

194. Gray SM, Wharf Higgins J, Rhodes RE. Understanding Physical Activity Motivation and Behavior Through Self-Determination and Servant Leadership Theories in a Feasibility Study. *J Aging Phys Act.* 2018;26(3):419-429.

195. Greenlee H, Lew DL, Hershman DL, et al. Phase II Feasibility Study of a Weight Loss Intervention in Female Breast and Colorectal Cancer Survivors (SWOG S1008). *Obesity.* 2018;26(10):1539-1549.

196. Greenlee HA, Crew KD, Mata JM, et al. A Pilot Randomized Controlled Trial of a Commercial Diet and Exercise Weight Loss Program in Minority Breast Cancer Survivors. *Obesity.* 2013;21(1):65-76.

197. GreenMills LL, Davison KK, Gordon KE, Li K, Jurkowski JM. Evaluation of a childhood obesity awareness campaign targeting head start families: designed by parents for parents. *J Health Care Poor Underserved.* 2013;24(2 Suppl):25-33.

198. Gribble LS, Falciglia G, Davis AM, Couch SC. A curriculum based on social learning theory emphasizing fruit exposure and positive parent child-feeding strategies: a pilot study. *J Am Diet Assoc.* 2003;103(1):100-103.

199. Griffin T, Sun Y, Sidhu M, et al. Healthy Dads, Healthy Kids UK, a weight management programme for fathers: feasibility RCT. *BMJ Open.* 2019;9(12):e033534.

200. Grim M, Hortz B, Petosa R. Impact evaluation of a pilot web-based intervention to increase physical activity. *Am J Health Promot.* 2011;25(4):227-230.

201. Grimes A, Baker M. The Effects of a Citywide Bike Share System on Active Transportation Among College Students: A Randomized Controlled Pilot Study. *Health Educ Behav.* 2020;47(3):412-418.

202. Gross CR, Kreitzer MJ, Russas V, Treesak C, Frazier PA, Hertz MI. Mindfulness meditation to reduce symptoms after organ transplant: a pilot study. *Altern Ther Health Med.* 2004;10(3):58-66.

203. Guldan GS, Fan HC, Ma X, Ni ZZ, Xiang X, Tang MZ. Culturally appropriate nutrition education improves infant feeding and growth in rural Sichuan, China. *J Nutr.* 2000;130(5):1204-1211.

204. Hageman PA, Walker SN, Pullen CH. Tailored versus standard internet-delivered interventions to promote physical activity in older women. *J Geriatr Phys Ther.* 2005;28(1):28-33.

205. Haile C, Kirk A, Cogan N, Janssen X, Gibson AM, MacDonald B. Pilot Testing of a Nudge-Based Digital Intervention (Welbot) to Improve Sedentary Behaviour and Wellbeing in the Workplace. *Int J Environ Res Public Health.* 2020;17(16).

206. Haines J, Douglas S, Mirotta JA, et al. Guelph Family Health Study: pilot study of a home-based obesity prevention intervention. *Canadian Journal of Public Health-Revue Canadienne De Sante Publique.* 2018;109(4):549-560.

207. Hamilton-Reeves JM, Johnson CN, Hand LK, et al. Feasibility of a Weight Management Program Tailored for Overweight Men with Localized Prostate Cancer - A Pilot Study. *Nutr Cancer.* 2020:1-16.

208. Han MK, Cho B, Kwon H, et al. A Mobile-Based Comprehensive Weight Reduction Program for the Workplace (Health-On): Development and Pilot Study. *JMIR Mhealth Uhealth.* 2019;7(11):e11158.

209. Hanna SM, Walker PW, Walker JF, et al. A Smoking Cessation Project for African American Women: Implications for Relational Research. *Families, Systems and Health.* 2003;21(4):383-395.

210. Harrington RA, Scarborough P, Hodgkins C, et al. A Pilot Randomized Controlled Trial of a Digital Intervention Aimed at Improving Food Purchasing Behavior: The Front-of-Pack Food Labels Impact on Consumer Choice Study. *JMIR Form Res.* 2019;3(2):e9910.

211. Harris KM, Schiele SE, Emery CF. Pilot randomized trial of brief behavioral treatment for insomnia in patients with heart failure. *Heart Lung.* 2019;48(5):373-380.

212. Harvey AG, Farrell C. The efficacy of a Pennebaker-like writing intervention for poor sleepers. *Behav Sleep Med.* 2003;1(2):115-124.

213. Harvey-Berino J, Pintauro SJ, Gold EC. The feasibility of using Internet support for the maintenance of weight loss. *Behav Modif.* 2002;26(1):103-116.

214. Harvey-Berino J, Pope L, Gold BC, Leonard H, Belliveau C. Undergrad and Overweight: An Online Behavioral Weight Management Program for College Students. *Journal of Nutrition Education and Behavior.* 2012;44(6):604-608.

215. Harvey-Berino J, Rourke J. Obesity prevention in preschool native-american children: a pilot study using home visiting. *Obes Res.* 2003;11(5):606-611.

216. Hawkes AL, Patrao TA, Green A, Aitken JF. CanPrevent: a telephone-delivered intervention to reduce multiple behavioural risk factors for colorectal cancer. *BMC Cancer.* 2012;12:560.

217. Hawley SR, Beckman H, Bishop T. Development of an obesity prevention and management program for children and adolescents in a rural setting. *J Community Health Nurs.* 2006;23(2):69-80.

218. Heideman WH, de Wit M, Middelkoop BJ, et al. DiAlert: a prevention program for overweight first degree relatives of type 2 diabetes patients: results of a pilot study to test feasibility and acceptability. *Trials.* 2012;13:178.

219. Hergenroeder AL, Gibbs BB, Kotlarczyk MP, et al. Sit Less for Successful Aging Pilot Study: Feasibility of an Intervention to Reduce Sedentary Time in Older Adults in Independent Living Communities. *Cardiopulm Phys Ther J.* 2020;31(4):142-151.

220. Herman A, Nelson BB, Teutsch C, Chung PJ. "Eat Healthy, Stay Active!": a coordinated intervention to improve nutrition and physical activity among Head Start parents, staff, and children. *Am J Health Promot.* 2012;27(1):e27-36.

221. Hoch CC, Reynolds CF, 3rd, Buysse DJ, et al. Protecting sleep quality in later life: a pilot study of bed restriction and sleep hygiene. *J Gerontol B Psychol Sci Soc Sci.* 2001;56(1):P52-59.

222. Hoeger KM, Kochman L, Wixom N, Craig K, Miller RK, Guzick DS. A randomized, 48-week, placebo-controlled trial of intensive lifestyle modification and/or metformin therapy in overweight women with polycystic ovary syndrome: a pilot study. *Fertil Steril.* 2004;82(2):421-429.

223. Hollis-Hansen K, Seidman J, O'Donnell S, Epstein LH. Mothers' DASH diet adherence and food purchases after week-long episodic future thinking intervention. *Appetite.* 2020;154:104757.

224. Hopkins LC, Fristad M, Goodway JD, et al. Feasibility and acceptability of technology-based caregiver engagement strategies delivered in a summertime childhood obesity prevention intervention: results from an internal pilot of the Camp NERF (Nutrition, Education, Recreation, and Fitness) study. *Pilot Feasibility Stud.* 2018;4:153.

225. Hotz C, Gibson RS. Participatory nutrition education and adoption of new feeding practices are associated with improved adequacy of complementary diets among rural Malawian children: a pilot study. *Eur J Clin Nutr.* 2005;59(2):226-237.

226. Hsu YT, Buckworth J, Focht BC, O'Connell AA. Feasibility of a Self-Determination Theory-based exercise intervention promoting Healthy at Every Size with sedentary overweight women: Project CHANGE. *Psychology of Sport and Exercise.* 2013;14(2):283-292.

227. Huang JJ, Lin HS, Yen M, Kan WM, Lee BO, Chen CH. Effects of a workplace multiple cardiovascular disease risks reduction program. *Asian Nurs Res (Korean Soc Nurs Sci).* 2013;7(2):74-82.

228. Huang RC, Silva D, Beilin L, et al. Feasibility of conducting an early pregnancy diet and lifestyle e-health intervention: the Pregnancy Lifestyle Activity Nutrition (PLAN) project. *J Dev Orig Health Dis.* 2020;11(1):58-70.

229. Huberty JL, Siahpush M, Beighle A, Fuhrmeister E, Silva P, Welk G. Ready for recess: a pilot study to increase physical activity in elementary school children. *J Sch Health.* 2011;81(5):251-257.

230. Hui AL, Ludwig SM, Gardiner P, et al. Community-based exercise and dietary intervention during pregnancy: A pilot study. *Canadian Journal of Diabetes.* 2006;30(2):169-175.

231. Hunt MK, Lobb R, Delichatsios HK, Stone C, Emmons K, Gillman MW. Process evaluation of a clinical preventive nutrition intervention. *Prev Med.* 2001;33(2 Pt 1):82-90.

232. Husband CJ, Wharf-Higgins J, Rhodes RE. A feasibility randomized trial of an identity-based physical activity intervention among university students. *Health Psychol Behav Med.* 2019;7(1):128-146.

233. Hyman DJ, Ho KS, Dunn JK, Simons-Morton D. Dietary intervention for cholesterol reduction in public clinic patients. *Am J Prev Med.* 1998;15(2):139-145.

234. Innerd AL, Azevedo LB, Batterham AM. The effect of a curriculum-based physical activity intervention on accelerometer-assessed physical activity in schoolchildren: A non-randomised mixed methods controlled before-and-after study. *PLoS One.* 2019;14(12):e0225997.

235. Insull Jr W, Henderson MM, Prentice RL, et al. Results of a randomized feasibility study of a low-fat diet. *Archives of Internal Medicine.* 1990;150(2):421-427.

236. Islam NS, Wyatt LC, Patel SD, et al. Evaluation of a community health worker pilot intervention to improve diabetes management in Bangladeshi immigrants with type 2 diabetes in New York City. *Diabetes Educ.* 2013;39(4):478-493.

237. Islam NS, Zanowiak JM, Wyatt LC, et al. A randomized-controlled, pilot intervention on diabetes prevention and healthy lifestyles in the New York City Korean community. *J Community Health.* 2013;38(6):1030-1041.

238. Jaber LA, Pinelli NR, Brown MB, et al. Feasibility of group lifestyle intervention for diabetes prevention in Arab Americans. *Diabetes Res Clin Pract.* 2011;91(3):307-315.

239. Jacobson D, Melnyk BM. A primary care healthy choices intervention program for overweight and obese school-age children and their parents. *J Pediatr Health Care.* 2012;26(2):126-138.

240. Jago R, Sebire SJ, Cooper AR, et al. Bristol girls dance project feasibility trial: outcome and process evaluation results. *Int J Behav Nutr Phys Act.* 2012;9:83.

241. Janicke DM, Gray WN, Mathews AE, et al. A pilot study examining a group-based behavioral family intervention for obese children enrolled in medicaid: Differential outcomes by race. *Children's Health Care.* 2011;40(3):212-231.

242. Jantz C, Anderson J, Gould SM. Using computer-based assessments to evaluate interactive multimedia nutrition education among low-income predominantly Hispanic participants. *J Nutr Educ Behav.* 2002;34(5):252-260.

243. Jayne J. Incorporation of Edutainment Into Intervention and Evaluation: The Jump With Jill (JWJ) Program. *Front Public Health.* 2019;7:163.

244. Jeffery RW, Pirie PL, Rosenthal BS, Gerber WM, Murray DM. Nutrition education in supermarkets: an unsuccessful attempt to influence knowledge and product sales. *J Behav Med.* 1982;5(2):189-200.

245. Jenkins I, Djuric Z, Darga L, DiLaura NM, Magnan M, Hryniuk WM. Relationship of psychiatric diagnosis and weight loss maintenance in obese breast cancer survivors. *Obes Res.* 2003;11(11):1369-1375.

246. Jenkinson KA, Naughton G, Benson AC. The GLAMA (Girls! Lead! Achieve! Mentor! Activate!) physical activity and peer leadership intervention pilot project: a process evaluation using the RE-AIM framework. *BMC Public Health.* 2012;12:55.

247. Jensen GL, Roy MA, Buchanan AE, Berg MB. Weight loss intervention for obese older women: improvements in performance and function. *Obes Res.* 2004;12(11):1814-1820.

248. Johansson L, Hagman E, Danielsson P. A novel interactive mobile health support system for pediatric obesity treatment: a randomized controlled feasibility trial. *BMC Pediatr.* 2020;20(1):447.

249. John JC, Wang J, McNeill LHM, et al. A Mixed Methods Study on Engagement and Satisfaction with a Digitally-Enhanced Pilot Intervention Among African American and Hispanic Women. *J Immigr Minor Health.* 2020.

250. Johns LJ, Dihigo S, Gilder RE. Collaborative Effort to Manage Childhood Obesity: Parental Employer, Health Insurance, and Employee Health Clinic. *J Dr Nurs Pract.* 2018;11(1):35-42.

251. Johnston B, El-Arabi A, Tuomela K, Nelson D. The Food Doctors: A pilot study to connect urban children and medical students using nutrition education. *Health Education Journal.* 2019;78(4):441-450.

252. Johnston V, Gane EM, Brown W, et al. Feasibility and impact of sit-stand workstations with and without exercise in office workers at risk of low back pain: A pilot comparative effectiveness trial. *Appl Ergon.* 2019;76:82-89.

253. Jones CHD, Owens JA, Pham B. Can a brief educational intervention improve parents' knowledge of healthy children's sleep? A pilot-test. *Health Education Journal.* 2013;72(5):601-610.

254. Jones RA, Riethmuller A, Hesketh K, Trezise J, Batterham M, Okely AD. Promoting fundamental movement skill development and physical activity in early childhood settings: a cluster randomized controlled trial. *Pediatr Exerc Sci.* 2011;23(4):600-615.

255. Joshi PP, Quintiliani LM, McCarthy AC, et al. A Randomized Controlled Feasibility Trial in Behavioral Weight Management for Underserved Postpartum African American Women: The RENEW Study. *Prev Chronic Dis.* 2018;15:E77.

256. Joyner D, Wengreen H, Aguilar S, Madden G. Effects of the FIT Game on Physical Activity in Sixth Graders: A Pilot Reversal Design Intervention Study. *JMIR Serious Games.* 2019;7(2):e13051.

257. Kaddumukasa M, Nakibuuka J, Mugenyi L, et al. Feasibility study of a targeted self-management intervention for reducing stroke risk factors in a high-risk population in Uganda. *J Neurol Sci.* 2018;386:23-28.

258. Kalarchian MA, Levine MD, Marcus MD. Structured Dietary Interventions in the Treatment of Severe Pediatric Obesity: A Pilot Study. *Bariatr Surg Pract Patient Care.* 2013;8(2):58-60.

259. Kaminsky LA, Jones J, Riggin K, Strath SJ. A pedometer-based physical activity intervention for patients entering a maintenance cardiac rehabilitation program: a pilot study. *Cardiovasc Diagn Ther.* 2013;3(2):73-79.

260. Katzmarzyk PT, Champagne CM, Tudor-Locke C, et al. A short-term physical activity randomized trial in the Lower Mississippi Delta. *PLoS One.* 2011;6(10):e26667.

261. Keating R, Ahern S, Bisgood L, Mernagh K, Nicolson GH, Barrett EM. Stand up, stand out. Feasibility of an active break targeting prolonged sitting in university students. *J Am Coll Health.* 2020:1-7.

262. Keaver L, Yiannakou I, Zhang FF. Integrating Nutrition into Outpatient Oncology Care-A Pilot Trial of the NutriCare Program. *Nutrients.* 2020;12(11).

263. Kelder S, Hoelscher DM, Barroso CS, Walker JL, Cribb P, Hu S. The CATCH Kids Club: a pilot after-school study for improving elementary students' nutrition and physical activity. *Public Health Nutr.* 2005;8(2):133-140.

264. Kelleher CC, Fallon UB, McCarthy E, et al. Feasibility of a lifestyle cardiovascular health promotion programme for 8-15-year-olds in Irish general practice: Results of the Galway Health Project. *Health Promotion International.* 1999;14(3):221-229.

265. Kelly A, Arjunan P, van der Ploeg HP, Rissel C, Borg J, Wen LM. The implementation of a pilot playground markings project in four Australian primary schools. *Health Promot J Austr.* 2012;23(3):183-187.

266. Kennedy BM, Paeratakul S, Champagne CM, et al. A pilot church-based weight loss program for African-American adults using church members as health educators: a comparison of individual and group intervention. *Ethn Dis.* 2005;15(3):373-378.

267. Khoshnevisan F, Kimiagar M, Kalantaree N, Valaee N, Shaheedee N. Effect of nutrition education and diet modification in iron depleted preschool children in nurseries in Tehran: a pilot study. *Int J Vitam Nutr Res.* 2004;74(4):264-268.

268. Kilanowski JF, Lin L. Effects of a healthy eating intervention on Latina migrant farmworker mothers. *Fam Community Health.* 2013;36(4):350-362.

269. Kim C, Draska M, Hess ML, Wilson EJ, Richardson CR. A web-based pedometer programme in women with a recent history of gestational diabetes. *Diabet Med.* 2012;29(2):278-283.

270. Kim H, Song HJ, Han HR, Kim KB, Kim MT. Translation and validation of the dietary approaches to stop hypertension for koreans intervention: culturally tailored dietary guidelines for Korean Americans with high blood pressure. *J Cardiovasc Nurs.* 2013;28(6):514-523.

271. Kim SH, Shin MS, Lee HS, et al. Randomized pilot test of a simultaneous stage-matched exercise and diet intervention for breast cancer survivors. *Oncol Nurs Forum.* 2011;38(2):E97-106.

272. King A, Harris AP, Perez LM, Wilund KR. A workplace wellness program results in improvements in physical activity and blood pressure in the staff of a hemodialysis clinic. *Journal of the American Society of Nephrology.* 2020;31:844.

273. Kok MSY, Bryant L, Cook C, Blackmore S, Jones M. Integrating Local Knowledge into a National Programme: Evidence from a Community-Based Diabetes Prevention Education Programme. *Healthcare (Basel).* 2019;7(1).

274. Kolden GG, Strauman TJ, Ward A, et al. A pilot study of group exercise training (GET) for women with primary breast cancer: feasibility and health benefits. *Psychooncology.* 2002;11(5):447-456.

275. Kolodziejczyk JK, Norman GJ, Barrera-Ng A, et al. Feasibility and effectiveness of an automated bilingual text message intervention for weight loss: pilot study. *JMIR Res Protoc.* 2013;2(2):e48.

276. Kong AS, Sussman AL, Yahne C, Skipper BJ, Burge MR, Davis SM. School-based health center intervention improves body mass index in overweight and obese adolescents. *J Obes.* 2013;2013:575016.

277. Kosma M, Cardinal BJ, McCubbin JA. A pilot study of a web-based physical activity motivational program for adults with physical disabilities. *Disabil Rehabil.* 2005;27(23):1435-1442.

278. Kotler LA, Fischer Etu S, Davies M, Devlin MJ, Attia E, Walsh BT. An open trial of an intensive summer day treatment program for severely overweight adolescents. *Eating and Weight Disorders.* 2006;11(4):e119-e122.

279. Koutoukidis DA, Beeken RJ, Manchanda R, et al. Diet, physical activity, and health-related outcomes of endometrial cancer survivors in a behavioral lifestyle program: the Diet and Exercise in Uterine Cancer Survivors (DEUS) parallel randomized controlled pilot trial. *Int J Gynecol Cancer.* 2019;29(3):531-540.

280. Kozey-Keadle S, Libertine A, Staudenmayer J, Freedson P. The Feasibility of Reducing and Measuring Sedentary Time among Overweight, Non-Exercising Office Workers. *J Obes.* 2012;2012:282303.

281. Kramer MK, Vanderwood KK, Arena VC, et al. Evaluation of a Diabetes Prevention Program Lifestyle Intervention in Older Adults: A Randomized Controlled Study in Three Senior/Community Centers of Varying Socioeconomic Status. *Diabetes Educ.* 2018;44(2):118-129.

282. Kreisel K. Evaluation of a computer-based nutrition education tool. *Public Health Nutr.* 2004;7(2):271-277.

283. Kreuzfeld S, Preuss M, Weippert M, Stoll R. Health effects and acceptance of a physical activity program for older long-term unemployed workers. *Int Arch Occup Environ Health.* 2013;86(1):99-105.

284. Krystia O, Ambrose T, Darlington G, Ma DWL, Buchholz AC, Haines J. A randomized home-based childhood obesity prevention pilot intervention has favourable effects on parental body composition: preliminary evidence from the Guelph Family Health Study. *BMC Obes.* 2019;6:10.

285. Kubo A, Kurtovich E, McGinnis M, et al. A Randomized Controlled Trial of mHealth Mindfulness Intervention for Cancer Patients and Informal Cancer Caregivers: A Feasibility Study Within an Integrated Health Care Delivery System. *Integr Cancer Ther.* 2019;18:1534735419850634.

286. Kulick D, Langer RD, Ashley JM, Gans KM, Schlauch K, Feller C. Live well: a practical and effective low-intensity dietary counseling intervention for use in primary care patients with dyslipidemia--a randomized controlled pilot trial. *BMC Fam Pract.* 2013;14:59.

287. Kumanyika SK, Obarzanek E, Robinson TN, Beech BM. Phase 1 of the Girls health Enrichment Multi-site Studies (GEMS): conclusion. *Ethn Dis.* 2003;13(1 Suppl 1):S88-91.

288. Kumar S, Croghan IT, Biggs BK, et al. Family-Based Mindful Eating Intervention in Adolescents with Obesity: A Pilot Randomized Clinical Trial. *Children (Basel).* 2018;5(7).

289. Kwon BC, VanDam C, Chiuve SE, et al. Improving Heart Disease Risk Through Quality-Focused Diet Logging: Pre-Post Study of a Diet Quality Tracking App. *JMIR Mhealth Uhealth.* 2020;8(12):e21733.

290. Lai A, Stewart S, Wan A, et al. Development and feasibility of a brief Zero-time Exercise intervention to reduce sedentary behaviour and enhance physical activity: A pilot trial. *Health Soc Care Community.* 2019;27(4):e233-e245.

291. Lai AYK, Stewart SM, Wan ANT, et al. Training to implement a community program has positive effects on health promoters: JC FAMILY Project. *Transl Behav Med.* 2018;8(6):838-850.

292. Lalonde L, Gray-Donald K, Lowensteyn I, et al. Comparing the benefits of diet and exercise in the treatment of dyslipidemia. *Prev Med.* 2002;35(1):16-24.

293. Lane H, Porter KJ, Hecht E, Harris P, Kraak V, Zoellner J. Kids SIPsmartER: A Feasibility Study to Reduce Sugar-Sweetened Beverage Consumption Among Middle School Youth in Central Appalachia. *American Journal of Health Promotion.* 2018;32(6):1386-1401.

294. Laranjo L, Quiroz JC, Tong HL, Arevalo Bazalar M, Coiera E. A Mobile Social Networking App for Weight Management and Physical Activity Promotion: Results From an Experimental Mixed Methods Study. *J Med Internet Res.* 2020;22(12):e19991.

295. Larsen B, Benitez T, Cano M, et al. Web-Based Physical Activity Intervention for Latina Adolescents: Feasibility, Acceptability, and Potential Efficacy of the Niñas Saludables Study. *J Med Internet Res.* 2018;20(5):e170.

296. Lau EY, Lau PW, Chung PK, Ransdell LB, Archer E. Evaluation of an Internet-short message service-based intervention for promoting physical activity in Hong Kong Chinese adolescent school children: a pilot study. *Cyberpsychol Behav Soc Netw.* 2012;15(8):425-434.

297. Laws RA, Denney-Wilson EA, Taki S, et al. Key Lessons and Impact of the Growing Healthy mHealth Program on Milk Feeding, Timing of Introduction of Solids, and Infant Growth: Quasi-Experimental Study. *Journal of Medical Internet Research.* 2018;20(4):1-1.

298. Leach HJ, Potter KB, Hidde MC. A Group Dynamics-Based Exercise Intervention to Improve Physical Activity Maintenance in Breast Cancer Survivors. *J Phys Act Health.* 2019;16(9):785-791.

299. Leahey TM, Wing RR. A randomized controlled pilot study testing three types of health coaches for obesity treatment: Professional, peer, and mentor. *Obesity (Silver Spring).* 2013;21(5):928-934.

300. Leahy AA, Eather N, Smith JJ, et al. Feasibility and Preliminary Efficacy of a Teacher-Facilitated High-Intensity Interval Training Intervention for Older Adolescents. *Pediatr Exerc Sci.* 2019;31(1):107-117.

301. Lear SA, Ignaszewski A, Laquer EA, Pritchard PH, Frohlich JJ. Extensive lifestyle management intervention following cardiac rehabilitation: pilot study. *Rehabil Nurs.* 2001;26(6):227-232.

302. LeCheminant JD, Smith JD, Covington NK, Hardin-Renschen T, Heden T. Pedometer use in university freshmen: a randomized controlled pilot study. *Am J Health Behav.* 2011;35(6):777-784.

303. Lee AH, Jancey J, Howat P, Burke L, Kerr DA, Shilton T. Effectiveness of a home-based postal and telephone physical activity and nutrition pilot program for seniors. *J Obes.* 2011;2011.

304. Lee S, Schorr E, Chi CL, Treat-Jacobson D, Mathiason MA, Lindquist R. Peer Group and Text Message-Based Weight-Loss and Management Intervention for African American Women. *West J Nurs Res.* 2018;40(8):1203-1219.

305. Legault C, Jennings JM, Katula JA, et al. Designing clinical trials for assessing the effects of cognitive training and physical activity interventions on cognitive outcomes: the Seniors Health and Activity Research Program Pilot (SHARP-P) study, a randomized controlled trial. *BMC Geriatr.* 2011;11:27.

306. Leung LY, Chan AW, Sit JW, Liu T, Taylor-Piliae RE. Tai Chi in Chinese adults with metabolic syndrome: A pilot randomized controlled trial. *Complement Ther Med.* 2019;46:54-61.

307. Levin GT, Greenwood KM, Singh F, Newton RU. Modality of exercise influences rate of decrease in depression for cancer survivors with elevated depressive symptomatology. *Support Care Cancer.* 2018;26(5):1597-1606.

308. Levin S, Martin MW, McKenzie TL, DeLouise AC. Assessment of a pilot video's effect on physical activity and heart health for young children. *Fam Community Health.* 2002;25(3):10-17.

309. Levine E, Olander C, Lefebvre C, Cusick P, Biesiadecki L, McGoldrick D. The Team Nutrition pilot study: lessons learned from implementing a comprehensive school-based intervention. *J Nutr Educ Behav.* 2002;34(2):109-116.

310. Levine MD, Ringham RM, Kalarchian MA, Wisniewski L, Marcus MD. Is family-based behavioral weight control appropriate for severe pediatric obesity? *Int J Eat Disord.* 2001;30(3):318-328.

311. Lew MS, L'Allemand D, Meli D, et al. Evaluating a childhood obesity program with the Reach, Effectiveness, Adoption, Implementation, Maintenance (RE-AIM) framework. *Prev Med Rep.* 2019;13:321-326.

312. Lewis BA, Martinson BC, Sherwood NE, Avery MD. A pilot study evaluating a telephone-based exercise intervention for pregnant and postpartum women. *J Midwifery Womens Health.* 2011;56(2):127-131.

313. Lindberg NM, Stevens VJ, Vega-López S, Kauffman TL, Calderón MR, Cervantes MA. A weight-loss intervention program designed for Mexican-American women: cultural adaptations and results. *J Immigr Minor Health.* 2012;14(6):1030-1039.

314. Linde JA, Jeffery RW. Testing a brief self-directed behavioral weight control program. *Behav Med.* 2011;37(2):47-53.

315. Ling J, Robbins LB, Zhang N, et al. Using Facebook in a Healthy Lifestyle Intervention: Feasibility and Preliminary Efficacy. *West J Nurs Res.* 2018;40(12):1818-1842.

316. Link LB, Thompson SM, Bosland MC, Lumey LH. Adherence to a low-fat diet in men with prostate cancer. *Urology.* 2004;64(5):970-975.

317. Linnan LA, Reiter PL, Duffy C, Hales D, Ward DS, Viera AJ. Assessing and promoting physical activity in African American barbershops: results of the FITStop pilot study. *Am J Mens Health.* 2011;5(1):38-46.

318. Liou TH, Chen CH, Hsu CY, Chou P, Chiu HW. A pilot study of videoconferencing for an Internet-based weight loss programme for obese adults in Taiwan. *J Telemed Telecare.* 2006;12(7):370-373.

319. Lipscombe LL, Delos-Reyes F, Glenn AJ, et al. The Avoiding Diabetes After Pregnancy Trial in Moms Program: Feasibility of a Diabetes Prevention Program for Women With Recent Gestational Diabetes Mellitus. *Can J Diabetes.* 2019;43(8):613-620.

320. Locher I, Waselewski M, Sonneville K, Resnicow K, Chang T. Grocery Delivery of Healthy Foods to Pregnant Young Women With Low Incomes: Feasibility and Acceptability Mixed Methods Study. *JMIR Form Res.* 2020;4(12):e21602.

321. Locher JL, Vickers KS, Buys DR, et al. A randomized controlled trial of a theoretically-based behavioral nutrition intervention for community elders: lessons learned from the Behavioral Nutrition Intervention for Community Elders Study. *J Acad Nutr Diet.* 2013;113(12):1675-1682.

322. Logue EE, Bourguet CC, Palmieri PA, et al. The better weight-better sleep study: a pilot intervention in primary care. *Am J Health Behav.* 2012;36(3):319-334.

323. Long JD, Armstrong ML, Amos E, et al. Pilot using World Wide Web to prevent diabetes in adolescents. *Clin Nurs Res.* 2006;15(1):67-79.

324. Low KG, Giasson H, Connors S, Freeman D, Weiss R. Testing the effectiveness of motivational interviewing as a weight reduction strategy for obese cardiac patients: a pilot study. *Int J Behav Med.* 2013;20(1):77-81.

325. Lubans DR, Morgan PJ, Aguiar EJ, Callister R. Randomized controlled trial of the Physical Activity Leaders (PALs) program for adolescent boys from disadvantaged secondary schools. *Prev Med.* 2011;52(3-4):239-246.

326. Lucumí DI, Sarmiento OL, Forero R, Gomez LF, Espinosa G. Community intervention to promote consumption of fruits and vegetables, smoke-free homes, and physical activity among home caregivers in Bogotá, Colombia. *Prev Chronic Dis.* 2006;3(4):A120.

327. Lynch E, Emery-Tiburcio E, Dugan S, et al. Results of ALIVE: A Faith-Based Pilot Intervention to Improve Diet Among African American Church Members. *Prog Community Health Partnersh.* 2019;13(1):19-30.

328. Ma Y, Olendzki BC, Chiriboga D, et al. PDA-assisted low glycemic index dietary intervention for type II diabetes: a pilot study. *Eur J Clin Nutr.* 2006;60(10):1235-1243.

329. Mabweazara SZ, Leach LL, Ley C, Smith M. A six week contextualised physical activity intervention for women living with HIV and AIDS of low socioeconomic status: a pilot study. *AIDS Care.* 2018;30(sup2):61-65.

330. Macdonell K, Brogan K, Naar-King S, Ellis D, Marshall S. A pilot study of motivational interviewing targeting weight-related behaviors in overweight or obese African American adolescents. *J Adolesc Health.* 2012;50(2):201-203.

331. Maciel Cancian AC, Schuster de Souza LA, Araujo Liboni RP, Machado WdL, Oliveira MdS. Effects of a dialectical behavior therapy-based skills group intervention for obese individuals: a Brazilian pilot study. *Eating and Weight Disorders-Studies on Anorexia Bulimia and Obesity.* 2019;24(6):1099-1111.

332. Maes L, Cook TL, Ottovaere C, et al. Pilot evaluation of the HELENA (Healthy Lifestyle in Europe by Nutrition in Adolescence) Food-O-Meter, a computer-tailored nutrition advice for adolescents: a study in six European cities. *Public Health Nutr.* 2011;14(7):1292-1302.

333. Maier IB, Stricker L, Ozel Y, Wagnerberger S, Bischoff SC, Bergheim I. A low fructose diet in the treatment of pediatric obesity: a pilot study. *Pediatr Int.* 2011;53(3):303-308.

334. Maloney AE, Stempel A, Wood ME, Patraitis C, Beaudoin C. Can Dance Exergames Boost Physical Activity as a School-Based Intervention? *Games Health J.* 2012;1(6):416-421.

335. Mama SK, Bhuiyan N, Bopp MJ, McNeill LH, Lengerich EJ, Smyth JM. A faith-based mind-body intervention to improve psychosocial well-being among rural adults. *Transl Behav Med.* 2020;10(3):546-554.

336. Mama SK, Bhuiyan N, Bopp MJ, McNeill LH, Lengerich EJ, Smyth JM. A faith-based minda-body intervention to improve psychosocial well-being among rural adults. *Translational Behavioral Medicine.* 2020;10(3):546-554.

337. Mantzari E, Galloway C, Wijndaele K, Brage S, Griffin SJ, Marteau TM. Impact of sit-stand desks at work on energy expenditure, sitting time and cardio-metabolic risk factors: Multiphase feasibility study with randomised controlled component. *Prev Med Rep.* 2019;13:64-72.

338. Marquez B, Wing RR. Feasibility of enlisting social network members to promote weight loss among Latinas. *J Acad Nutr Diet.* 2013;113(5):680-687.

339. Marra MV, Lilly CL, Nelson KR, Woofter DR, Malone J. A pilot randomized controlled trial of a telenutrition weight loss intervention in middle-aged and older men with multiple risk factors for cardiovascular disease. *Nutrients.* 2019;11(2).

340. Marsh S, Taylor R, Galland B, Gerritsen S, Parag V, Maddison R. Results of the 3 Pillars Study (3PS), a relationship-based programme targeting parent-child interactions, healthy lifestyle behaviours, and the home environment in parents of preschool-aged children: A pilot randomised controlled trial. *PLoS One.* 2020;15(9):e0238977.

341. Martens MP, Buscemi J, Smith AE, Murphy JG. The short-term efficacy of a brief motivational intervention designed to increase physical activity among college students. *J Phys Act Health.* 2012;9(4):525-532.

342. Martin K, Fontaine KR, Nicklas BJ, Dennis KE, Goldberg AP, Hochberg MC. Weight loss and exercise walking reduce pain and improve physical functioning in overweight postmenopausal women with knee osteoarthritis. *J Clin Rheumatol.* 2001;7(4):219-223.

343. Masini A, Marini S, Leoni E, et al. Active Breaks: A Pilot and Feasibility Study to Evaluate the Effectiveness of Physical Activity Levels in a School Based Intervention in an Italian Primary School. *Int J Environ Res Public Health.* 2020;17(12).

344. Maskarinec G, Chan CL, Meng L, Franke AA, Cooney RV. Exploring the feasibility and effects of a high-fruit and -vegetable diet in healthy women. *Cancer Epidemiol Biomarkers Prev.* 1999;8(10):919-924.

345. Matz-Costa C, Lubben J, Lachman ME, Lee H, Choi YJ. A Pilot Randomized Trial of an Intervention to Enhance the Health-Promoting Effects of Older Adults' Activity Portfolios: The Engaged4Life Program. *J Gerontol Soc Work.* 2018;61(8):792-816.

346. Mauriello LM, Driskell MM, Sherman KJ, Johnson SS, Prochaska JM, Prochaska JO. Acceptability of a school-based intervention for the prevention of adolescent obesity. *J Sch Nurs.* 2006;22(5):269-277.

347. Maxwell M, Lemacks J, Coccia C, Ralston PA, Ilich JZ. A student-led pilot project to improve calcium intake and a healthy lifestyle in African American Communities. *Topics in Clinical Nutrition.* 2012;27(1):54-66.

348. McCallum Z, Wake M, Gerner B, et al. Can Australian general practitioners tackle childhood overweight/obesity? Methods and processes from the LEAP (Live, Eat and Play) randomized controlled trial. *J Paediatr Child Health.* 2005;41(9-10):488-494.

349. McClure JB, Catz SL, Ludman EJ, Richards J, Riggs K, Grothaus L. Feasibility and acceptability of a multiple risk factor intervention: the Step Up randomized pilot trial. *BMC Public Health.* 2011;11:167.

350. McCurry SM, LaFazia DM, Pike KC, Logsdon RG, Teri L. Development and evaluation of a sleep education program for older adults with dementia living in adult family homes. *Am J Geriatr Psychiatry.* 2012;20(6):494-504.

351. McGaffey AL, Abatemarco DJ, Jewell IK, Fidler SK, Hughes K. Fitwits MD™: an office-based tool and games for conversations about obesity with 9- to 12-year-old children. *J Am Board Fam Med.* 2011;24(6):768-771.

352. McGrath Davis A, Sampilo M, Gayes LA, Smith C, Steele RG. The Effect of Visual Cues on Dietary Behavior: A Pilot Study. *Children's Health Care.* 2013;42(4):353-363.

353. McKay HG, King D, Eakin EG, Seeley JR, Glasgow RE. The diabetes network internet-based physical activity intervention: a randomized pilot study. *Diabetes Care.* 2001;24(8):1328-1334.

354. McKenzie SB, O'Connell J, Smith LA, Ottinger WE. A primary intervention program (pilot study) for Mexican American children at risk for type 2 diabetes. *Diabetes Educ.* 1998;24(2):180-187.

355. McKibbin CL, Patterson TL, Norman G, et al. A lifestyle intervention for older schizophrenia patients with diabetes mellitus: A randomized controlled trial. *Schizophrenia Research.* 2006;86(1-3):36-44.

356. McTiernan A, Ulrich C, Kumai C, et al. Anthropometric and hormone effects of an eight-week exercise-diet intervention in breast cancer patients: results of a pilot study. *Cancer Epidemiol Biomarkers Prev.* 1998;7(6):477-481.

357. Memon AR, Masood T, Awan WA, Waqas A. The effectiveness of an incentivized physical activity programme (Active Student) among female medical students in Pakistan: A Randomized Controlled Trial. *J Pak Med Assoc.* 2018;68(10):1438-1445.

358. Menza M, Vreeland B, Minsky S, Gara M, Radler DR, Sakowitz M. Managing atypical antipsychotic-associated weight gain: 12-month data on a multimodal weight control program. *J Clin Psychiatry.* 2004;65(4):471-477.

359. Messier SP, Loeser RF, Mitchell MN, et al. Exercise and weight loss in obese older adults with knee osteoarthritis: a preliminary study. *J Am Geriatr Soc.* 2000;48(9):1062-1072.

360. Mier N, Tanguma J, Millard AV, Villarreal EK, Alen M, Ory MG. A pilot walking program for Mexican-American women living in colonias at the border. *Am J Health Promot.* 2011;25(3):172-175.

361. Militão AG, Karnikowski MGO, da Silva FR, Militão ESG, Pereira RMS, Campbell CSG. Effects of a recreational physical activity and healthy habits orientation program, using an illustrated diary, on the cardiovascular risk profile of overweight and obese schoolchildren: A pilot study in a public school in Brasilia, Federal District, Brazil. *Diabetes, Metabolic Syndrome and Obesity: Targets and Therapy.* 2013;6:445-451.

362. Miller CK, Kristeller JL, Headings A, Nagaraja H, Miser WF. Comparative effectiveness of a mindful eating intervention to a diabetes self-management intervention among adults with type 2 diabetes: a pilot study. *J Acad Nutr Diet.* 2012;112(11):1835-1842.

363. Min J, Tan Z, Abadie L, Townsend S, Xue H, Wang Y. An Evaluation of the Effectiveness of the National Aeronautics and Space Administration Mission-X Child Health Promotion Program in the United States. *Am J Health Promot.* 2018;32(6):1333-1339.

364. Minett MM, Binkley TL, Holm RP, Runge M, Specker BL. Feasibility and Effects on Muscle Function of an Exercise Program for Older Adults. *Med Sci Sports Exerc.* 2020;52(2):441-448.

365. Moeller LV, Lindhardt CL, Andersen MS, Glintborg D, Ravn P. Motivational interviewing in obese women with polycystic ovary syndrome - a pilot study. *Gynecol Endocrinol.* 2019;35(1):76-80.

366. Moens E, Braet C. Training parents of overweight children in parenting skills: a 12-month evaluation. *Behav Cogn Psychother.* 2012;40(1):1-18.

367. Montagni I, Dehman A, Yu Z, et al. Effectiveness of a Blended Web-Based Intervention to Raise Sleep Awareness at Workplace: The WarmUapp™ Pilot Study. *J Occup Environ Med.* 2019;61(6):e253-e259.

368. Moore SM, Charvat JM. Using the CHANGE intervention to enhance long-term exercise. *Nurs Clin North Am.* 2002;37(2):273-283, vi.

369. Morey MC, Ekelund C, Pearson M, et al. Project LIFE: a partnership to increase physical activity in elders with multiple chronic illnesses. *J Aging Phys Act.* 2006;14(3):324-343.

370. Morgan PJ, Collins CE, Plotnikoff RC, et al. Efficacy of a workplace-based weight loss program for overweight male shift workers: the Workplace POWER (Preventing Obesity Without Eating like a Rabbit) randomized controlled trial. *Prev Med.* 2011;52(5):317-325.

371. Morgan PJ, Lubans DR, Callister R, et al. The 'Healthy Dads, Healthy Kids' randomized controlled trial: efficacy of a healthy lifestyle program for overweight fathers and their children. *Int J Obes (Lond).* 2011;35(3):436-447.

372. Morris AS, Murphy RC, Shepherd SO, Healy GN, Edwardson CL, Graves LEF. A multi-component intervention to sit less and move more in a contact centre setting: a feasibility study. *BMC Public Health.* 2019;19(1):292.

373. Morrison R, Reilly JJ, Penpraze V, et al. Children, parents and pets exercising together (CPET): exploratory randomised controlled trial. *BMC Public Health.* 2013;13:1096.

374. Moy ML, Weston NA, Wilson EJ, Hess ML, Richardson CR. A pilot study of an Internet walking program and pedometer in COPD. *Respir Med.* 2012;106(9):1342-1350.

375. Mutrie N, Doolin O, Fitzsimons CF, et al. Increasing older adults' walking through primary care: results of a pilot randomized controlled trial. *Fam Pract.* 2012;29(6):633-642.

376. Nanduri AP, Fullman S, Morell L, Buyske S, Wagner ML. Pilot Study for Implementing an Osteoporosis Education and Exercise Program in an Assisted Living Facility and Senior Community. *J Appl Gerontol.* 2018;37(6):745-762.

377. Napolitano MA, Hayes S, Bennett GG, Ives AK, Foster GD. Using Facebook and text messaging to deliver a weight loss program to college students. *Obesity (Silver Spring).* 2013;21(1):25-31.

378. Narayan KM, Hoskin M, Kozak D, et al. Randomized clinical trial of lifestyle interventions in Pima Indians: a pilot study. *Diabet Med.* 1998;15(1):66-72.

379. Natovich R, Gayus N, Azmon M, et al. A Comprehensive Intervention for Promoting Successful Aging Amongst Older People With Diabetes With Below-Normal Cognitive Function-A Feasibility Study. *Front Endocrinol (Lausanne).* 2020;11:348.

380. Nepper MJ, McAtee JR, Wheeler L, Chai W. Mobile Phone Text Message Intervention on Diabetes Self-Care Activities, Cardiovascular Disease Risk Awareness, and Food Choices among Type 2 Diabetes Patients. *Nutrients.* 2019;11(6).

381. Neumark-Sztainer D, Story M, Hannan PJ, Rex J. New Moves: a school-based obesity prevention program for adolescent girls. *Prev Med.* 2003;37(1):41-51.

382. Newton Jr RL, Perri MG. A randomized pilot trial of exercise promotion in sedentary African-American adults. *Ethnicity and Disease.* 2004;14(4):548-557.

383. Newton MJ, Hayes SC, Janda M, et al. Safety, feasibility and effects of an individualised walking intervention for women undergoing chemotherapy for ovarian cancer: a pilot study. *BMC Cancer.* 2011;11:389.

384. Niederhauser VP, Maddock J, LeDoux F, Arnold M. Building strong and ready Army families: a multirisk reduction health promotion pilot study. *Mil Med.* 2005;170(3):227-233.

385. Niemeier HM, Leahey T, Palm Reed K, Brown RA, Wing RR. An acceptance-based behavioral intervention for weight loss: a pilot study. *Behav Ther.* 2012;43(2):427-435.

386. Nies MA, Artinian NT, Schim SM, Vander Wal JS, Sherrick-Escamilla S. Effects of lay health educator interventions on activity, diet, and health risks in an urban Mexican American community. *Journal of Primary Prevention.* 2004;25(4):441-455.

387. Nishigaki M, Ota A, Kusakabe T, Matsuzaki C, Taguchi S, Kazuma K. Feasibility and efficiency of indirect lifestyle interventions in offspring of type 2 diabetic patients. *Public Health Genomics.* 2011;14(2):77-84.

388. Nobes JP, Langley SE, Klopper T, Russell-Jones D, Laing RW. A prospective, randomized pilot study evaluating the effects of metformin and lifestyle intervention on patients with prostate cancer receiving androgen deprivation therapy. *BJU Int.* 2012;109(10):1495-1502.

389. Nyberg G, Andermo S, Nordenfelt A, Lidin M, Hellénius ML. Effectiveness of a Family Intervention to Increase Physical Activity in Disadvantaged Areas-A Healthy Generation, a Controlled Pilot Study. *Int J Environ Res Public Health.* 2020;17(11).

390. O'Brien N, Roe C, Reeves S. A quantitative nutritional evaluation of a healthy eating intervention in primary school children in a socioeconomically disadvantaged area - A pilot study. *Health Education Journal.* 2002;61(4):320-328.

391. O'Connor TM, Beltran A, Musaad S, et al. Feasibility of Targeting Hispanic Fathers and Children in an Obesity Intervention: Papás Saludables Niños Saludables. *Child Obes.* 2020;16(6):379-392.

392. O'Connor TM, Hilmers A, Watson K, Baranowski T, Giardino AP. Feasibility of an obesity intervention for paediatric primary care targeting parenting and children: Helping HAND. *Child Care Health Dev.* 2013;39(1):141-149.

393. Oftedal S, Burrows T, Fenton S, Murawski B, Rayward AB, Duncan MJ. Feasibility and Preliminary Efficacy of an m-Health Intervention Targeting Physical Activity, Diet, and Sleep Quality in Shift-Workers. *International Journal of Environmental Research and Public Health.* 2019;16(20):18.

394. Ogden J, Maxwell H, Wong A. Development and feasibility study of an app (Ladle) for weight loss and behaviour change. *PeerJ.* 2019;7:e6907.

395. Okhomina VI, Seals SR, Anugu P, Adu-Boateng G, Sims M, Marshall GD, Jr. Adherence and retention of African Americans in a randomized controlled trial with a yoga-based intervention: the effects of health promoting programs on cardiovascular disease risk study. *Ethn Health.* 2020;25(6):812-824.

396. Olalla J, García de Lomas JM, Márquez E, et al. Experience of Using an App in HIV Patients Older Than 60 Years: Pilot Program. *JMIR Mhealth Uhealth.* 2019;7(3):e9904.

397. Oliver M, Schofield G, McEvoy E. An integrated curriculum approach to increasing habitual physical activity in children: a feasibility study. *J Sch Health.* 2006;76(2):74-79.

398. Ornes LL, Ransdell LB, Robertson L, Trunnell E, Moyer-Mileur L. A 6-month pilot study of effects of a physical activity intervention on life satisfaction with a sample of three generations of women. *Percept Mot Skills.* 2005;100(3 Pt 1):579-591.

399. Overgaard K, Nannerup K, Lunen MKB, Maindal HT, Larsen RG. Exercise more or sit less? A randomized trial assessing the feasibility of two advice-based interventions in obese inactive adults. *J Sci Med Sport.* 2018;21(7):708-713.

400. Owen MB, Kerner C, Taylor SL, et al. The Feasibility of a Novel School Peer-Led Mentoring Model to Improve the Physical Activity Levels and Sedentary Time of Adolescent Girls: The Girls Peer Activity (G-PACT) Project. *Children (Basel).* 2018;5(6).

401. Pagán-Ortiz ME, Cortés DE. Feasibility of an Online Health Intervention for Latinas With Chronic Pain. *Rehabilitation Psychology.* 2020.

402. Pagoto S, Tulu B, Agu E, Waring ME, Oleski JL, Jake-Schoffman DE. Using the Habit App for Weight Loss Problem Solving: Development and Feasibility Study. *JMIR Mhealth Uhealth.* 2018;6(6):e145.

403. Pahor M, Blair SN, Espeland M, et al. Effects of a physical activity intervention on measures of physical performance: Results of the lifestyle interventions and independence for elders pilot (LIFE-P) study. *Journals of Gerontology - Series A Biological Sciences and Medical Sciences.* 2006;61(11):1157-1165.

404. Parks EP, Moore RH, Li ZY, et al. Assessing the Feasibility of a Social Media to Promote Weight Management Engagement in Adolescents with Severe Obesity: Pilot Study. *JMIR Research Protocols.* 2018;7(3):10.

405. Paschali AA, Kalantzi-Azizi A, Goodrick GK, Papadatou D, Balasubramanyam A. Accelerometer feedback to promote physical activity in adults with type 2 diabetes: A pilot study. *Perceptual and Motor Skills.* 2005;100(1):61-68.

406. Patten CA, Vickers KS, Martin JE, Williams CD. Exercise interventions for smokers with a history of alcoholism: exercise adherence rates and effect of depression on adherence. *Addict Behav.* 2003;28(4):657-667.

407. Paul IM, Savage JS, Anzman SL, et al. Preventing obesity during infancy: a pilot study. *Obesity (Silver Spring).* 2011;19(2):353-361.

408. Pauley AM, Hohman E, Savage JS, et al. Gestational Weight Gain Intervention Impacts Determinants of Healthy Eating and Exercise in Overweight/Obese Pregnant Women. *Journal of Obesity.* 2018;2018:12.

409. Paxton RJ, Taylor WC, Hudnall GE, Christie J. Goal Setting to Promote a Health Lifestyle. *Int Proc Chem Biol Environ Eng.* 2012;39:101-105.

410. Pbert L, Druker S, Gapinski MA, et al. A school nurse-delivered intervention for overweight and obese adolescents. *J Sch Health.* 2013;83(3):182-193.

411. Pearson N, Biddle SJH, Griffiths P, Sherar LB, McGeorge S, Haycraft E. Reducing screen-time and unhealthy snacking in 9-11 year old children: the Kids FIRST pilot randomised controlled trial. *BMC Public Health.* 2020;20(1):122.

412. Pekmezi D, Ainsworth C, Holly T, et al. Physical Activity and Related Psychosocial Outcomes From a Pilot Randomized Trial of an Interactive Voice Response System-Supported Intervention in the Deep South. *Health Educ Behav.* 2018;45(6):957-966.

413. Pelletier JR, Nguyen M, Bradley K, Johnsen M, McKay C. A study of a structured exercise program with members of an ICCD certified clubhouse: Program design, benefits, and implications for feasibility. *Psychiatric Rehabilitation Journal.* 2005;29(2):89-96.

414. Perkiö-Mäkelä M, Notkola V, Husman K. Activities supporting work ability as a part of farmers' occupational health services. *Journal of Occupational Rehabilitation.* 1999;9(2):107-114.

415. Pfammatter AF, Marchese SH, Pellegrini C, Daly E, Davidson M, Spring B. Using the Preparation Phase of the Multiphase Optimization Strategy to Develop a Messaging Component for Weight Loss: Formative and Pilot Research. *JMIR Form Res.* 2020;4(5):e16297.

416. Pickett M, Mock V, Ropka ME, Cameron L, Coleman M, Podewils L. Adherence to moderate-intensity exercise during breast cancer therapy. *Cancer Pract.* 2002;10(6):284-292.

417. Pienaar AE, Du Toit D, Truter L. The effect of a multidisciplinary physical activity intervention on the body composition and physical fitness of obese children. *Journal of Sports Medicine and Physical Fitness.* 2013;53(4):415-427.

418. Pierce J, Legg S, Godfrey JR, Kawabata E. The effects of introducing electric adjustable height desks in an office setting on workplace physical activity levels: A randomised control field trial. *Work.* 2019;62(1):139-150.

419. Pinelli NR, Brown MB, Herman WH, Jaber LA. Family support is associated with success in achieving weight loss in a group lifestyle intervention for diabetes prevention in Arab Americans. *Ethn Dis.* 2011;21(4):480-484.

420. Pinto BM, Goldstein MG, DePue JD, Milan FB. Acceptability and feasibility of physician-based activity counseling. The PAL project. *Am J Prev Med.* 1998;15(2):95-102.

421. Plotnikoff RC, Pickering MA, Glenn N, et al. The effects of a supplemental, theory-based physical activity counseling intervention for adults with type 2 diabetes. *J Phys Act Health.* 2011;8(7):944-954.

422. Podgorski CA, Kessler K, Cacia B, Peterson DR, Henderson CM. Physical activity intervention for older adults with intellectual disability: report on a pilot project. *Ment Retard.* 2004;42(4):272-283.

423. Polonsky WH, Zee J, Yee MA, Crosson MA, Jackson RA. A community-based program to encourage patients' attention to their own diabetes care: pilot development and evaluation. *Diabetes Educ.* 2005;31(5):691-699.

424. Pope L, Garnett B, Dibble M. Lessons Learned Through the Implementation of an eHealth Physical Activity Gaming Intervention with High School Youth. *Games Health J.* 2018;7(2):136-142.

425. Porock D, Kristjanson LJ, Tinnelly K, Duke T, Blight J. An exercise intervention for advanced cancer patients experiencing fatigue: a pilot study. *J Palliat Care.* 2000;16(3):30-36.

426. Potter JD, Graves KL, Finnegan JR, et al. The Cancer and Diet Intervention Project: A community-based intervention to reduce nutrition-related risk of cancer. *Health Education Research.* 1990;5(4):489-503.

427. Powers SW, Byars KC, Mitchell MJ, Patton SR, Schindler T, Zeller MH. A Randomized Pilot Study of Behavioral Treatment to Increase Calorie Intake in Toddlers with Cystic Fibrosis. *Children's Health Care.* 2003;32(4):297-311.

428. Prince SA, Reed JL, Cotie LM, Harris J, Pipe AL, Reid RD. Results of the Sedentary Intervention Trial in Cardiac Rehabilitation (SIT-CR Study): A pilot randomized controlled trial. *Int J Cardiol.* 2018;269:317-324.

429. Ptomey LT, Vidoni ED, Montenegro-Montenegro E, et al. The Feasibility of Remotely Delivered Exercise Session in Adults With Alzheimer's Disease and Their Caregivers. *J Aging Phys Act.* 2019;27(5):670–677.

430. Quinn M. Introduction of active video gaming into the middle school curriculum as a school-based childhood obesity intervention. *J Pediatr Health Care.* 2013;27(1):3-12.

431. Quirk H, Glazebrook C, Blake H. A physical activity intervention for children with type 1 diabetes- steps to active kids with diabetes (STAK-D): a feasibility study. *BMC Pediatr.* 2018;18(1):37.

432. Ramsay J, Hoffmann A. Smoking cessation and relapse prevention among undergraduate students: a pilot demonstration project. *J Am Coll Health.* 2004;53(1):11-18.

433. Rankins J, Sampson W, Brown B, Jenkins-Salley T. Dietary Approaches to Stop Hypertension (DASH) intervention reduces blood pressure among hypertensive African American patients in a neighborhood health care center. *J Nutr Educ Behav.* 2005;37(5):259-264.

434. Ransdell LB, Dratt J, Kennedy C, O'Neill S, De Voe D. Daughters and mothers exercising together (DAMET): A 12-week pilot project designed to improve physical self-perception and increase recreational physical activity. *Women and Health.* 2001;33(3-4):101-116.

435. Ransdell LB, Robertson L, Ornes L, Moyer-Mileur L. Generations Exercising Together to Improve Fitness (GET FIT): a pilot study designed to increase physical activity and improve health-related fitness in three generations of women. *Women Health.* 2004;40(3):77-94.

436. Ransdell LB, Taylor A, Oakland D, Schmidt J, Moyer-Mileur L, Shultz B. Daughters and mothers exercising together: effects of home- and community-based programs. *Med Sci Sports Exerc.* 2003;35(2):286-296.

437. Raynor HA, Steeves EA, Bassett DR, Jr., Thompson DL, Gorin AA, Bond DS. Reducing TV watching during adult obesity treatment: two pilot randomized controlled trials. *Behav Ther.* 2013;44(4):674-685.

438. Read A, Ramwell H, Storer H, Webber J. A primary care intervention programme for obesity and coronary heart disease risk factor reduction. *Br J Gen Pract.* 2004;54(501):272-278.

439. Reale S, Kearney CM, Hetherington MM, et al. The Feasibility and Acceptability of Two Methods of Snack Portion Control in United Kingdom (UK) Preschool Children: Reduction and Replacement. *Nutrients.* 2018;10(10).

440. Reeves MM, Spark L, Hickman IJ, McCarthy N, Demark-Wahnefried W, Eakin EG. Feasibility of a weight loss intervention for women following treatment for breast cancer: Living Well after Breast Cancer. *Obesity Facts.* 2013;6:47.

441. Reilly RE, Cincotta M, Doyle J, et al. A pilot study of Aboriginal health promotion from an ecological perspective. *BMC Public Health.* 2011;11:749.

442. Reinhardt JA, van der Ploeg HP, Grzegrzulka R, Timperley JG. lmplementing lifestyle change through phone-based motivational interviewing in rural-based women with previous gestational diabetes mellitus. *Health Promot J Austr.* 2012;23(1):5-9.

443. Remmert JE, Woodworth A, Chau L, Schumacher LM, Butryn ML, Schneider M. Pilot Trial of an Acceptance-Based Behavioral Intervention to Promote Physical Activity Among Adolescents. *Journal of School Nursing.* 2019;35(6):449-461.

444. Resnick B. Testing the effect of the WALC intervention on exercise adherence in older adults. *J Gerontol Nurs.* 2002;28(6):40-49.

445. Resnicow K, Orlandi MA, Vaccaro D, Wynder E. Implementation of a pilot school-site cholesterol reduction intervention. *J Sch Health.* 1989;59(2):74-78.

446. Rhodes RE, Murray H, Temple VA, Tuokko H, Higgins JW. Pilot study of a dog walking randomized intervention: effects of a focus on canine exercise. *Prev Med.* 2012;54(5):309-312.

447. Richards EA, Woodcox S. A county extension-delivered, email-mediated walking intervention: A programme evaluation. *Health Education Journal.* 2018;77(5):615-624.

448. Richardson CR, Avripas SA, Neal DL, Marcus SM. Increasing lifestyle physical activity in patients with depression or other serious mental illness. *J Psychiatr Pract.* 2005;11(6):379-388.

449. Richardson CR, Brown BB, Foley S, Dial KS, Lowery JC. Feasibility of adding enhanced pedometer feedback to nutritional counseling for weight loss. *J Med Internet Res.* 2005;7(5):e56.

450. Riley KM, Glasgow RE, Eakin EG. Resources for Health: A Social-Ecological Intervention for Supporting Self-management of Chronic Conditions. *J Health Psychol.* 2001;6(6):693-705.

451. Rimmer JH, Nicola T, Riley B, Creviston T. Exercise training for African Americans with disabilities residing in difficult social environments. *Am J Prev Med.* 2002;23(4):290-295.

452. Rimmer JH, Silverman K, Braunschweig C, Quinn L, Liu Y. Feasibility of a health promotion intervention for a group of predominantly African American women with type 2 diabetes. *Diabetes Educ.* 2002;28(4):571-580.

453. Rioux J, Howerter A. Outcomes from a Whole-Systems Ayurvedic Medicine and Yoga Therapy Treatment for Obesity Pilot Study. *J Altern Complement Med.* 2019;25(S1):S124-s137.

454. Ritenbaugh C, Teufel-Shone NI, Aickin MG, et al. A lifestyle intervention improves plasma insulin levels among Native American high school youth. *Preventive Medicine.* 2003;36(3):309-319.

455. Robbins LB, Gretebeck KA, Kazanis AS, Pender NJ. Girls on the move program to increase physical activity participation. *Nurs Res.* 2006;55(3):206-216.

456. Robbins LB, Pfeiffer KA, Maier KS, Lo YJ, Wesolek Ladrig SM. Pilot intervention to increase physical activity among sedentary urban middle school girls: a two-group pretest-posttest quasi-experimental design. *J Sch Nurs.* 2012;28(4):302-315.

457. Robert McComb JJ, Tacon A, Randolph P, Caldera Y. A pilot study to examine the effects of a mindfulness-based stress-reduction and relaxation program on levels of stress hormones, physical functioning, and submaximal exercise responses. *J Altern Complement Med.* 2004;10(5):819-827.

458. Robinson KR, Long AL, Leighton P, et al. Chair based exercise in community settings: a cluster randomised feasibility study. *BMC Geriatr.* 2018;18(1):82.

459. Robinson SA, Bisson AN, Hughes ML, Ebert J, Lachman ME. Time for change: using implementation intentions to promote physical activity in a randomised pilot trial. *Psychol Health.* 2019;34(2):232-254.

460. Robinson TN, Killen JD, Kraemer HC, et al. Dance and reducing television viewing to prevent weight gain in African-American girls: the Stanford GEMS pilot study. *Ethn Dis.* 2003;13(1 Suppl 1):S65-77.

461. Rock CL, Byers TE, Colditz GA, et al. Reducing breast cancer recurrence with weight loss, a vanguard trial: The Exercise and Nutrition to Enhance Recovery and Good Health for You (ENERGY) Trial. *Contemporary Clinical Trials.* 2013;34(2):282-295.

462. Rosal MC, Lemon SC, Nguyen OH, Driscoll NE, Ditaranto L. Translation of the diabetes prevention program lifestyle intervention for promoting postpartum weight loss among low-income women. *Transl Behav Med.* 2011;1(4):530-538.

463. Rosal MC, Olendzki B, Reed GW, Gumieniak O, Scavron J, Ockene I. Diabetes self-management among low-income Spanish-speaking patients: a pilot study. *Ann Behav Med.* 2005;29(3):225-235.

464. Rotheram-Borus MJ, Tomlinson M, Gwegwe M, Comulada WS, Kaufman N, Keim M. Diabetes buddies: peer support through a mobile phone buddy system. *Diabetes Educ.* 2012;38(3):357-365.

465. Rustad C, Smith C. Nutrition knowledge and associated behavior changes in a holistic, short-term nutrition education intervention with low-income women. *J Nutr Educ Behav.* 2013;45(6):490-498.

466. Saelens BE, Sallis JF, Wilfley DE, Patrick K, Cella JA, Buchta R. Behavioral weight control for overweight adolescents initiated in primary care. *Obes Res.* 2002;10(1):22-32.

467. Saksvig BI, Gittelsohn J, Harris SB, Hanley AJG, Valente TW, Zinman B. A pilot school-based healthy eating and physical activity intervention improves diet, food knowledge, and self-efficacy for native Canadian children. *Journal of Nutrition.* 2005;135(10):2392-2398.

468. Samaan Z, Schulze KM, Middleton C, et al. South Asian Heart Risk Assessment (SAHARA): Randomized Controlled Trial Design and Pilot Study. *Journal of Medical Internet Research.* 2013;15(8):1-1.

469. Samuel-Hodge CD, Johnston LF, Gizlice Z, et al. A pilot study comparing two weight loss maintenance interventions among low-income, mid-life women. *BMC Public Health.* 2013;13:653.

470. Sánchez-Johnsen LAP, Stolley MR, Fitzgibbon ML. Diet, physical activity, and breast health intervention for latina women. *Hispanic Health Care International.* 2006;4(2):101-110.

471. Sandlund M, Waterworth EL, Häger C. Using motion interactive games to promote physical activity and enhance motor performance in children with cerebral palsy. *Dev Neurorehabil.* 2011;14(1):15-21.

472. Sauder KA, Dabelea D, Bailey-Callahan R, et al. Targeting risk factors for type 2 diabetes in American Indian youth: the Tribal Turning Point pilot study. *Pediatr Obes.* 2018;13(5):321-329.

473. Savage JS, Adams EL, Rollins BY, Bleser JA, Marini ME. Teaching families to manage intake of candy in the home: Results from a feasibility study using multiphase optimization strategy (MOST). *Obes Sci Pract.* 2020;6(6):649-659.

474. Saxe GA, Major JM, Nguyen JY, Freeman KM, Downs TM, Salem CE. Potential attenuation of disease progression in recurrent prostate cancer with plant-based diet and stress reduction. *Integr Cancer Ther.* 2006;5(3):206-213.

475. Scheid A, Dyer NL, Dusek JA, Khalsa SBS. A Yoga-Based Program Decreases Physician Burnout in Neonatologists and Obstetricians at an Academic Medical Center. *Workplace Health Saf.* 2020;68(12):560-566.

476. Schleicher HE, Harris KJ, Campbell DG, Harrar SW. Mood management intervention for college smokers with elevated depressive symptoms: a pilot study. *J Am Coll Health.* 2012;60(1):37-45.

477. Schmutte T, Davidson L, O’Connell M. Improved Sleep, Diet, and Exercise in Adults with Serious Mental Illness: Results from a Pilot Self-Management Intervention. *Psychiatric Quarterly.* 2018;89(1):61-71.

478. Schneider JK, Mercer GT, Herning M, Smith CA, Prysak MD. Promoting exercise behavior in older adults: using a cognitive behavioral intervention. *J Gerontol Nurs.* 2004;30(4):45-53.

479. Schoeppe S, Salmon J, Williams SL, et al. Effects of an activity tracker and app intervention to increase physical activity in whole families—the step it up family feasibility study. *International Journal of Environmental Research and Public Health.* 2020;17(20):1-20.

480. Schwartz RP, Vitolins MZ, Case LD, et al. The YMCA Healthy, Fit, and Strong Program: a community-based, family-centered, low-cost obesity prevention/treatment pilot study. *Child Obes.* 2012;8(6):577-582.

481. Seal N, Seal J. Developing healthy childhood behaviour: outcomes of a summer camp experience. *Int J Nurs Pract.* 2011;17(4):428-434.

482. Sebire SJ, Banfield K, Jago R, et al. A process evaluation of the PLAN-A intervention (Peer-Led physical Activity iNtervention for Adolescent girls). *BMC Public Health.* 2019;19(1):1203.

483. Sedlak CA, Doheny MO, Estok PJ, Zeller RA. Tailored interventions to enhance osteoporosis prevention in women. *Orthop Nurs.* 2005;24(4):270-276; quiz 277.

484. Seguin RA, Perry CK, Solanki E, McCalmont JC, Ward JP, Jackson C. Mujeres Fuertes y Corazones Saludables, a Culturally Tailored Physical Activity and Nutrition Program for Rural Latinas: Findings from a Pilot Study. *Int J Environ Res Public Health.* 2019;16(4).

485. Self M, Brewer A, Kumanyika S, Doroshenko L, Carnaghi M, Brancato J. Pilot study to enhance start-up of a multicenter nutrition intervention trial. *J Am Diet Assoc.* 1998;98(3):322-325.

486. Shaibi GQ, Konopken Y, Hoppin E, Keller CS, Ortega R, Castro FG. Effects of a culturally grounded community-based diabetes prevention program for obese Latino adolescents. *Diabetes Educ.* 2012;38(4):504-512.

487. Sharp KJ, South CC, Chin Fatt C, Trivedi MH, Rethorst CD. Pilot Studies to Evaluate Feasibility of a Physical Activity Intervention for Persons With Depression. *J Sport Exerc Psychol.* 2020:1-9.

488. Sheedy J, Smith B, Bauman A, et al. A controlled trial of behavioural education to promote exercise among physiotherapy outpatients. *Aust J Physiother.* 2000;46(4):281-289.

489. Sherry AP, Pearson N, Ridgers ND, et al. Impacts of a Standing Desk Intervention within an English Primary School Classroom: A Pilot Controlled Trial. *Int J Environ Res Public Health.* 2020;17(19).

490. Shi-Chang X, Xin-Wei Z, Shui-Yang X, et al. Creating health-promoting schools in China with a focus on nutrition. *Health Promot Int.* 2004;19(4):409-418.

491. Shvedko AV, Thompson JL, Greig CA, Whittaker AC. Physical Activity Intervention for Loneliness (PAIL) in community-dwelling older adults: a randomised feasibility study. *Pilot Feasibility Stud.* 2020;6:73.

492. Siahpush M, Huberty JL, Beighle A. Does the effect of a school recess intervention on physical activity vary by gender or race? Results from the Ready for Recess pilot study. *J Public Health Manag Pract.* 2012;18(5):416-422.

493. Silveira DS, Barbosa Ferreira Lemos LFG, Tassitano RM, et al. Effect of a pilot multi-component intervention on motor performance and metabolic risks in overweight/obese youth. *Journal of sports sciences.* 2018;36(20):2317-2326.

494. Simmons D, Fleming C, Voyle J, Fou F, Feo S, Gatland B. A pilot urban church-based programme to reduce risk factors for diabetes among Western Samoans in New Zealand. *Diabet Med.* 1998;15(2):136-142.

495. Sin MK, Belza B, Logerfo J, Cunningham S. Evaluation of a community-based exercise program for elderly Korean immigrants. *Public Health Nurs.* 2005;22(5):407-413.

496. Sjöling M, Lundberg K, Englund E, Westman A, Jong MC. Effectiveness of motivational interviewing and physical activity on prescription on leisure exercise time in subjects suffering from mild to moderate hypertension. *BMC Res Notes.* 2011;4:352.

497. Skelly AH, Carlson JR, Leeman J, Holditch-Davis D, Soward ACM. Symptom-focused management for African American women with Type 2 diabetes: A pilot study. *Applied Nursing Research.* 2005;18(4):213-220.

498. Slusser W, Frankel F, Robison K, Fischer H, Cumberland WG, Neumann C. Pediatric overweight prevention through a parent training program for 2-4 year old Latino children. *Child Obes.* 2012;8(1):52-59.

499. Slusser WM, Sharif MZ, Erausquin JT, Kinsler JJ, Collin D, Prelip ML. Improving overweight among at-risk minority youth: results of a pilot intervention in after-school programs. *J Health Care Poor Underserved.* 2013;24(2 Suppl):12-24.

500. Small L, Bonds-McClain D, Vaughan L, Melnyk B, Gannon A, Thompson S. A parent-directed portion education intervention for young children: Be Beary Healthy. *J Spec Pediatr Nurs.* 2012;17(4):312-320.

501. Smith AL, Hoza B, Linnea K, et al. Pilot physical activity intervention reduces severity of ADHD symptoms in young children. *J Atten Disord.* 2013;17(1):70-82.

502. Smith DE, Heckemeyer CM, Kratt PP, Mason DA. Motivational interviewing to improve adherence to a behavioral weight-control program for older obese women with NIDDM. A pilot study. *Diabetes Care.* 1997;20(1):52-54.

503. Sniehotta FF, Dombrowski SU, Avenell A, et al. Randomised controlled feasibility trial of an evidence-informed behavioural intervention for obese adults with additional risk factors. *PLoS One.* 2011;6(8):e23040.

504. Song M, Lee CS, Lyons KS, Stoyles S, Winters-Stone KM. Assessing the feasibility of parent participation in a commercial weight loss program to improve child body mass index and weight-related health behaviors. *SAGE Open Med.* 2018;6:2050312118801220.

505. Spadola CE, Rottapel RE, Zhou ES, et al. A sleep hygiene and yoga intervention conducted in affordable housing communities: Pilot study results and lessons for a future trial. *Complement Ther Clin Pract.* 2020;39:101121.

506. Speck BJ, Looney SW. Effects of a minimal intervention to increase physical activity in women: daily activity records. *Nurs Res.* 2001;50(6):374-378.

507. Stark LJ, Spear S, Boles R, et al. A pilot randomized controlled trial of a clinic and home-based behavioral intervention to decrease obesity in preschoolers. *Obesity (Silver Spring).* 2011;19(1):134-141.

508. Stathi A, Withall J, Thompson JL, et al. Feasibility Trial Evaluation of a Peer Volunteering Active Aging Intervention: ACE (Active, Connected, Engaged). *Gerontologist.* 2020;60(3):571-582.

509. Steeves JA, Bassett DR, Fitzhugh EC, Raynor HA, Thompson DL. Can sedentary behavior be made more active? A randomized pilot study of TV commercial stepping versus walking. *International Journal of Behavioral Nutrition and Physical Activity.* 2012;9.

510. Stefanich CA, Witmer JM, Young BD, et al. Development, adaptation, and implementation of a cardiovascular health program for Alaska native women. *Health Promot Pract.* 2005;6(4):472-481.

511. Steger FL, Donnelly JE, Hull HR, Li X, Hu J, Sullivan DK. Intermittent and continuous energy restriction result in similar weight loss, weight loss maintenance, and body composition changes in a 6 month randomized pilot study. *Clinical Obesity.* 2020.

512. Steinberg DM, Levine EL, Askew S, Foley P, Bennett GG. Daily text messaging for weight control among racial and ethnic minority women: randomized controlled pilot study. *J Med Internet Res.* 2013;15(11):e244.

513. Stevens VJ, Corrigan SA, Obarzanek E, et al. Weight loss intervention in phase 1 of the Trials of Hypertension Prevention. The TOHP Collaborative Research Group. *Arch Intern Med.* 1993;153(7):849-858.

514. Stolley MR, Fitzgibbon ML, Wells A, Martinovich Z. Addressing multiple breast cancer risk factors in African-American women. *Journal of the National Medical Association.* 2004;96(1):76-86.

515. Story M, Lytle LA, Birnbaum AS, Perry CL. Peer-led, school-based nutrition education for young adolescents: feasibility and process evaluation of the TEENS study. *J Sch Health.* 2002;72(3):121-127.

516. Story M, Sherwood NE, Himes JH, et al. An after-school obesity prevention program for African-American girls: the Minnesota GEMS pilot study. *Ethn Dis.* 2003;13(1 Suppl 1):S54-64.

517. Stovitz SD, VanWormer JJ, Center BA, Bremer KL. Pedometers as a means to increase ambulatory activity for patients seen at a family medicine clinic. *J Am Board Fam Pract.* 2005;18(5):335-343.

518. Strath SJ, Swartz AM, Parker SJ, Miller NE, Grimm EK, Cashin SE. A pilot randomized controlled trial evaluating motivationally matched pedometer feedback to increase physical activity behavior in older adults. *J Phys Act Health.* 2011;8 Suppl 2(0 2):S267-274.

519. Stremler R, Hodnett E, Lee K, et al. A behavioral-educational intervention to promote maternal and infant sleep: a pilot randomized, controlled trial. *Sleep.* 2006;29(12):1609-1615.

520. Sweat V, Bruzzese JM, Albert S, Pinero DJ, Fierman A, Convit A. The Banishing Obesity and Diabetes in Youth (BODY) Project: description and feasibility of a program to halt obesity-associated disease among urban high school students. *J Community Health.* 2012;37(2):365-371.

521. Sweeney BM, Leigh Signal T, Babbage DR. Effect of a behavioral-educational sleep intervention for first-time mothers and their infants: Pilot of a controlled trial. *Journal of Clinical Sleep Medicine.* 2020;16(8):1265-1274.

522. Sweitzer SJ, Briley ME, Roberts-Gray C, et al. Psychosocial outcomes of lunch is in the bag, a parent program for packing healthful lunches for preschool children. *Journal of Nutrition Education and Behavior.* 2011;43(6):536-542.

523. Tan E, Healey D, Gray AR, Galland BC. Sleep hygiene intervention for youth aged 10 to 18 years with problematic sleep: a before-after pilot study. *BMC Pediatr.* 2012;12:189.

524. Tarro L, Llauradó E, Aceves-Martins M, et al. Impact of a youth-led social marketing intervention run by adolescents to encourage healthy lifestyles among younger school peers (EYTO-Kids project): a parallel-cluster randomised controlled pilot study. *J Epidemiol Community Health.* 2019;73(4):324-333.

525. Taylor L, Whittington F, Hollingsworth C, et al. Assessing the effectiveness of a walking program on physical function of residents living in an assisted living facility. *J Community Health Nurs.* 2003;20(1):15-26.

526. Teufel NI, Perry CL, Story M, et al. Pathways family intervention for third-grade American Indian children. *Am J Clin Nutr.* 1999;69(4 Suppl):803s-809s.

527. Tewary S, Cook N, Pandya N, McCurry SM. Pilot test of a six-week group delivery caregiver training program to reduce sleep disturbances among older adults with dementia (Innovative practice). *Dementia (London).* 2018;17(2):234-243.

528. Thomas EA, Mijangos JL, Hansen PA, et al. Mindfulness-Oriented Recovery Enhancement Restructures Reward Processing and Promotes Interoceptive Awareness in Overweight Cancer Survivors: Mechanistic Results From a Stage 1 Randomized Controlled Trial. *Integr Cancer Ther.* 2019;18:1534735419855138.

529. Thompson DA, Joshi A, Hernandez RG, et al. Nutrition education via a touchscreen: a randomized controlled trial in Latino immigrant parents of infants and toddlers. *Acad Pediatr.* 2012;12(5):412-419.

530. Thompson WM, Berry D, Hu J. A church-based intervention to change attitudes about physical activity among Black adolescent girls: a feasibility study. *Public Health Nurs.* 2013;30(3):221-230.

531. Trinh L, Wilson R, Williams HM, Sum AJ, Naylor PJ. Physicians promoting physical activity using pedometers and community partnerships: a real world trial. *Br J Sports Med.* 2012;46(4):284-290.

532. Trout KK, McGrath J, Flanagan J, Costello MC, Frey JC. A pilot study to increase fruit and vegetable intake in pregnant latina women. *J Prim Care Community Health.* 2012;3(1):2-5.

533. Tsai AG, Felton S, Hill JO, Atherly AJ. A randomized pilot trial of a full subsidy vs. a partial subsidy for obesity treatment. *Obesity (Silver Spring).* 2012;20(9):1838-1843.

534. Tse ACY. Brief Report: Impact of a Physical Exercise Intervention on Emotion Regulation and Behavioral Functioning in Children with Autism Spectrum Disorder. *J Autism Dev Disord.* 2020;50(11):4191-4198.

535. Tudor-Locke CE, Myers AM, Bell RC, Harris SB, Wilson Rodger N. Preliminary outcome evaluation of the First Step Program: a daily physical activity intervention for individuals with type 2 diabetes. *Patient Educ Couns.* 2002;47(1):23-28.

536. Tully C, Mackey E, Aronow L, et al. Parenting Intervention to Improve Nutrition and Physical Activity for Preschoolers with Type 1 Diabetes: A Feasibility Study. *J Pediatr Health Care.* 2018;32(6):548-556.

537. Tully MA, Cupples ME. UNISTEP (university students exercise and physical activity) study: a pilot study of the effects of accumulating 10,000 steps on health and fitness among university students. *J Phys Act Health.* 2011;8(5):663-667.

538. Turner J, Hayes S, Reul-Hirche H. Improving the physical status and quality of life of women treated for breast cancer: a pilot study of a structured exercise intervention. *J Surg Oncol.* 2004;86(3):141-146.

539. Upham K, Auer BJ, Sciamanna CN, et al. Adults Want to Play Too: Feasibility of an Adult Physical Activity Program Designed to Maximize Enjoyment. *J Phys Act Health.* 2020;17(2):230-235.

540. Uusitupa M, Louheranta A, Lindström J, et al. The Finnish Diabetes Prevention Study. *Br J Nutr.* 2000;83 Suppl 1:S137-142.

541. Vaitkevicius PV, Ebersold C, Shah MS, et al. Effects of aerobic exercise training in community-based subjects aged 80 and older: a pilot study. *J Am Geriatr Soc.* 2002;50(12):2009-2013.

542. Valentiner LS, Thorsen IK, Kongstad MB, et al. Effect of ecological momentary assessment, goal-setting and personalized phone-calls on adherence to interval walking training using the InterWalk application among patients with type 2 diabetes-A pilot randomized controlled trial. *PLoS One.* 2019;14(1):e0208181.

543. Valle CG, Tate DF, Mayer DK, Allicock M, Cai JW. A randomized trial of a Facebook-based physical activity intervention for young adult cancer survivors. *Journal of Cancer Survivorship.* 2013;7(3):355-368.

544. Van Blarigan EL, Chan H, Van Loon K, et al. Self-monitoring and reminder text messages to increase physical activity in colorectal cancer survivors (Smart Pace): a pilot randomized controlled trial. *BMC Cancer.* 2019;19(1):218.

545. van Doorn-van Atten MN, Haveman-Nies A, Heery D, de Vries JHM, de Groot L. Feasibility and Effectiveness of Nutritional Telemonitoring for Home Care Clients: A Pilot Study. *Gerontologist.* 2019;59(1):158-166.

546. van Horn LV, Stumbo P, Moag-Stahlberg A, et al. The Dietary Intervention Study in Children (DISC): dietary assessment methods for 8- to 10-year-olds. *J Am Diet Assoc.* 1993;93(12):1396-1403.

547. van Sluijs EM, van Poppel MN, Stalman WA, van Mechelen W. Feasibility and acceptability of a physical activity promotion programme in general practice. *Fam Pract.* 2004;21(4):429-436.

548. Vandelanotte C, De Bourdeaudhuij I. Acceptability and feasibility of a computer-tailored physical activity intervention using stages of change: project FAITH. *Health Educ Res.* 2003;18(3):304-317.

549. Vanhelst J, Fardy PS, Mikulovic J, et al. Changes in obesity, cardiorespiratory fitness and habitual physical activity following a one-year intervention program in obese youth: a pilot study. *J Sports Med Phys Fitness.* 2011;51(4):670-675.

550. Vann LH, Stanford FC, Durkin MW, Hanna A, Knight LM, Stallworth JR. "Moving and losing": A pilot study incorporating physical activity to decrease obesity in the pediatric population. *J S C Med Assoc.* 2013;109(4):116-120.

551. Varela Mato V, Caddick N, King JA, et al. The Impact of a Novel Structured Health Intervention for Truckers (SHIFT) on Physical Activity and Cardiometabolic Risk Factors. *J Occup Environ Med.* 2018;60(4):368-376.

552. Verloigne M, Bere E, Van Lippevelde W, et al. The effect of the UP4FUN pilot intervention on objectively measured sedentary time and physical activity in 10-12 year old children in Belgium: the ENERGY-project. *BMC Public Health.* 2012;12:805.

553. Vidmar AP, Pretlow R, Borzutzky C, et al. An addiction model-based mobile health weight loss intervention in adolescents with obesity. *Pediatr Obes.* 2019;14(2):e12464.

554. Villalba JA, Amirehsani K, Lewis TF. Increasing healthy behaviors in adolescents of Mexican heritage in rural emerging Latino communities: results from a school-based health intervention pilot study. *J Immigr Minor Health.* 2011;13(3):629-632.

555. von Gruenigen VE, Frasure HE, Kavanagh MB, Lerner E, Waggoner SE, Courneya KS. Feasibility of a lifestyle intervention for ovarian cancer patients receiving adjuvant chemotherapy. *Gynecol Oncol.* 2011;122(2):328-333.

556. Walch TJ, Rosenkranz RR, Schenkelberg MA, Fees BS, Dzewaltowski DA. Parent adoption and implementation of obesity prevention practices through building children's asking skills at family child care homes. *Eval Program Plann.* 2020;80:101810.

557. Wald ER, Ewing LJ, Moyer SCL, Eickhoff JC. An Interactive Web-Based Intervention to Achieve Healthy Weight in Young Children. *Clin Pediatr (Phila).* 2018;57(5):547-557.

558. Walker EA, Weiss L, Gary-Webb TL, et al. Power Up for Health: Pilot Study Outcomes of a Diabetes Prevention Program for Men from Disadvantaged Neighborhoods. *Am J Mens Health.* 2018;12(4):989-997.

559. Wan Yunus F, Tan XZ, Romli MH. Investigating the Feasibility of Exergame on Sleep and Emotion Among University Students. *Games Health J.* 2020;9(6):415-424.

560. Wang X, Hsu FC, Isom S, et al. Effects of a 12-month physical activity intervention on prevalence of metabolic syndrome in elderly men and women. *J Gerontol A Biol Sci Med Sci.* 2012;67(4):417-424.

561. Wang YJ, Boehmke M, Wu YW, Dickerson SS, Fisher N. Effects of a 6-week walking program on Taiwanese women newly diagnosed with early-stage breast cancer. *Cancer Nurs.* 2011;34(2):E1-13.

562. Wang YT, Taylor L, Pearl M, Chang LS. Effects of Tai Chi exercise on physical and mental health of college students. *Am J Chin Med.* 2004;32(3):453-459.

563. Warren JM, Henry CJ, Lightowler HJ, Bradshaw SM, Perwaiz S. Evaluation of a pilot school programme aimed at the prevention of obesity in children. *Health Promot Int.* 2003;18(4):287-296.

564. Warren KR, Ball MP, Feldman S, Liu F, McMahon RP, Kelly DL. Exercise Program Adherence Using a 5-Kilometer (5K) Event as an Achievable Goal in People With Schizophrenia. *Biological research for nursing.* 2011;13(4):383-390.

565. Waters DL, Vawter R, Qualls C, Chode S, Armamento-Villareal R, Villareal DT. Long-term maintenance of weight loss after lifestyle intervention in frail, obese older adults. *Journal of Nutrition, Health and Aging.* 2012:1-5.

566. Watson A, Timperio A, Brown H, Hesketh KD. Process evaluation of a classroom active break (ACTI-BREAK) program for improving academic-related and physical activity outcomes for students in years 3 and 4. *BMC Public Health.* 2019;19(1):633.

567. Watson N, Milat AJ, Thomas M, Currie J. The feasibility and effectiveness of pram walking groups for postpartum women in western Sydney. *Health Promot J Austr.* 2005;16(2):93-99.

568. Weaver RG, Webster CA, Egan C, Campos CMC, Michael RD, Vazou S. Partnerships for Active Children in Elementary Schools: Outcomes of a 2-Year Pilot Study to Increase Physical Activity During the School Day. *Am J Health Promot.* 2018;32(3):621-630.

569. Weber M, Wyne K. A cognitive/behavioral group intervention for weight loss in patients treated with atypical antipsychotics. *Schizophr Res.* 2006;83(1):95-101.

570. Wei C, Candler T, Crowne E, Hamilton-Shield JP. Interval Outcomes of a Lifestyle Weight-Loss Intervention in Early Adolescence. *Children (Basel).* 2018;5(6).

571. Welsh D, Lennie TA, Marcinek R, et al. Low-sodium diet self-management intervention in heart failure: pilot study results. *Eur J Cardiovasc Nurs.* 2013;12(1):87-95.

572. Wen LM, Orr N, Bindon J, Rissel C. Promoting active transport in a workplace setting: evaluation of a pilot study in Australia. *Health Promot Int.* 2005;20(2):123-133.

573. Wengle JG, Hamilton JK, Manlhiot C, et al. The 'Golden Keys' to health - a healthy lifestyle intervention with randomized individual mentorship for overweight and obesity in adolescents. *Paediatr Child Health.* 2011;16(8):473-478.

574. Wengreen HJ, Madden GJ, Aguilar SS, Smits RR, Jones BA. Incentivizing Children's Fruit and Vegetable Consumption: Results of a United States Pilot Study of the Food Dudes Program. *Journal of Nutrition Education and Behavior.* 2013;45(1):54-59.

575. West JS, Guelfi KJ, Dimmock JA, Jackson B. Testing the Feasibility and Preliminary Efficacy of an 8-Week Exercise and Compensatory Eating Intervention. *Nutrients.* 2018;10(7).

576. Whitley MD, Payán DD, Flórez KR, et al. Feasibility and acceptability of a mobile messaging program within a church-based healthy living intervention for African Americans and Latinos. *Health Informatics J.* 2020;26(2):880-896.

577. Whittemore R, Melkus GD, Sullivan A, Grey M. A nurse-coaching intervention for women with type 2 diabetes. *Diabetes Educ.* 2004;30(5):795-804.

578. Wick K, Faude O, Manes S, Zahner L, Donath L. I Can Stand Learning: A Controlled Pilot Intervention Study on the Effects of Increased Standing Time on Cognitive Function in Primary School Children. *Int J Environ Res Public Health.* 2018;15(2).

579. Williams JM, Power T, Stoneham J, DeGreg N, Siegel RM. A Feasibility Study of a Fit Kit School-Based Intervention to Improve the Health of Students and Their Families. *Reports.* 2020;3(1):7.

580. Wilson AJ, Jung ME, Cramp A, Simatovic J, Prapavessis H, Clarson C. Effects of a group-based exercise and self-regulatory intervention on obese adolescents' physical activity, social cognitions, body composition and strength: a randomized feasibility study. *J Health Psychol.* 2012;17(8):1223-1237.

581. Wilson DB, Porter JS, Parker G, Kilpatrick J. Anthropometric changes using a walking intervention in African American breast cancer survivors: a pilot study. *Prev Chronic Dis.* 2005;2(2):A16.

582. Wilson MG, Padilla HM, Meng L, Daniel CN. Impact of a workplace holiday weight gain prevention program. *Nutr Health.* 2019;25(3):173-177.

583. Wilson RW, Jacobsen PB, Fields KK. Pilot study of a home-based aerobic exercise program for sedentary cancer survivors treated with hematopoietic stem cell transplantation. *Bone Marrow Transplant.* 2005;35(7):721-727.

584. Wilson RW, Taliaferro LA, Jacobsen PB. Pilot study of a self-administered stress management and exercise intervention during chemotherapy for cancer. *Support Care Cancer.* 2006;14(9):928-935.

585. Witmer JM, Hensel MR, Holck PS, Ammerman AS, Will JC. Heart disease prevention for Alaska Native women: a review of pilot study findings. *J Womens Health (Larchmt).* 2004;13(5):569-578.

586. Wong RSM, Yu EYT, Wong TW, et al. Development and pilot evaluation of a mobile app on parent-child exercises to improve physical activity and psychosocial outcomes of Hong Kong Chinese children. *BMC Public Health.* 2020;20(1):1544.

587. Woods D, Leavey G, Meek R, Breslin G. Developing mental health awareness and help seeking in prison: a feasibility study of the State of Mind Sport programme. *Int J Prison Health.* 2020;16(4):403-416.

588. Wrieden WL, Symon A. The development and pilot evaluation of a nutrition education intervention programme for pregnant teenage women (food for life). *J Hum Nutr Diet.* 2003;16(2):67-71.

589. Wright JA, Phillips BD, Watson BL, Newby PK, Norman GJ, Adams WG. Randomized trial of a family-based, automated, conversational obesity treatment program for underserved populations. *Obesity (Silver Spring).* 2013;21(9):E369-378.

590. Wyse R, Wolfenden L, Campbell E, et al. A pilot study of a telephone-based parental intervention to increase fruit and vegetable consumption in 3-5-year-old children. *Public health nutrition.* 2011;14(12):2245-2253.

591. Xu M, Chattopadhyay K, Li JL, et al. Weight Management Programme for Overweight and Obese Adults in Ningbo, China: A Feasibility Pre- and Post-intervention Study. *Frontiers in public health.* 2019;7:5.

592. Yang CL, Chen CH. Effectiveness of aerobic gymnastic exercise on stress, fatigue, and sleep quality during postpartum: A pilot randomized controlled trial. *Int J Nurs Stud.* 2018;77:1-7.

593. Ye Q, Hu GY, Cai YB, et al. Structural exercise-based intervention for health problems in individuals with autism spectrum disorders: a pilot study. *Eur Rev Med Pharmacol Sci.* 2019;23(10):4313-4320.

594. Yeary KH, Cornell CE, Turner J, et al. Feasibility of an evidence-based weight loss intervention for a faith-based, rural, African American population. *Prev Chronic Dis.* 2011;8(6):A146.

595. Yoong SL, Grady A, Stacey F, et al. A pilot randomized controlled trial examining the impact of a sleep intervention targeting home routines on young children's (3-6 years) physical activity. *Pediatr Obes.* 2019;14(4):e12481.

596. Young L, Anderson J, Beckstrom L, Bellows L, Johnson SL. Using social marketing principles to guide the development of a nutrition education initiative for preschool-aged children. *J Nutr Educ Behav.* 2004;36(5):250-257.

597. Young PC, West SA, Ortiz K, Carlson J. A pilot study to determine the feasibility of the low glycemic index diet as a treatment for overweight children in primary care practice. *Ambul Pediatr.* 2004;4(1):28-33.

598. Zhang J, Jemmott Iii JB. Mobile App-Based Small-Group Physical Activity Intervention for Young African American Women: a Pilot Randomized Controlled Trial. *Prev Sci.* 2019;20(6):863-872.

599. Zhou YE, Buchowski MS, Akatue RA, Wu J, Liu J, Hargreaves MK. Physical Activity Levels and Cardiometabolic Risks in Obese African American Adults: A Pilot Intervention Study. *J Health Care Poor Underserved.* 2018;29(3):1027-1045.

600. Zoellner J, Hill JL, Grier K, et al. Randomized controlled trial targeting obesity-related behaviors: Better Together Healthy Caswell County. *Prev Chronic Dis.* 2013;10:E96.
