# Supplementary material for: Feasibility indicators in obesity-related behavioral intervention preliminary studies: a historical scoping review
Source: Pilot Feasibility Stud. 2023 Mar 22;9:46. doi: 10.1186/s40814-023-01270-w (PMC10032007; doi:10.1186/s40814-023-01270-w)
Supplement: Supplementary file 3 — Additional file 3: Supplementary File 3. PubMed Example Search Strategy. [file 40814_2023_1270_MOESM3_ESM.docx]

| Search number | Query | Sort By | Filters | Search Details |
| --- | --- | --- | --- | --- |
| 35 | (((pilot[Title/Abstract]) AND (obes*[Title/Abstract])) AND (screen[Title/Abstract])) AND (interven*[Title/Abstract]) | | from 2004 - 2020 | ("pilot"[Title/Abstract] AND "obes*"[Title/Abstract] AND "screen"[Title/Abstract] AND "interven*"[Title/Abstract]) AND (2004:2020[pdat]) |
| 34 | (((pilot[Title/Abstract]) AND (obes*[Title/Abstract])) AND (screen[Title/Abstract])) AND (interven*[Title/Abstract]) | | | "pilot"[Title/Abstract] AND "obes*"[Title/Abstract] AND "screen"[Title/Abstract] AND "interven*"[Title/Abstract] |
| 33 | (((feasibility[Title/Abstract]) AND (obes*[Title/Abstract])) AND (screen[Title/Abstract])) AND (interven*[Title/Abstract]) | | from 2006 - 2020 | ("feasibility"[Title/Abstract] AND "obes*"[Title/Abstract] AND "screen"[Title/Abstract] AND "interven*"[Title/Abstract]) AND (2006:2020[pdat]) |
| 32 | (((feasibility[Title/Abstract]) AND (obes*[Title/Abstract])) AND (screen[Title/Abstract])) AND (interven*[Title/Abstract]) | | | "feasibility"[Title/Abstract] AND "obes*"[Title/Abstract] AND "screen"[Title/Abstract] AND "interven*"[Title/Abstract] |
| 31 | (((pilot[Title/Abstract]) AND (obes*[Title/Abstract])) AND (fitness[Title/Abstract])) AND (interven*[Title/Abstract]) | | from 2001 - 2020 | ("pilot"[Title/Abstract] AND "obes*"[Title/Abstract] AND "fitness"[Title/Abstract] AND "interven*"[Title/Abstract]) AND (2001:2020[pdat]) |
| 30 | (((pilot[Title/Abstract]) AND (obes*[Title/Abstract])) AND (fitness[Title/Abstract])) AND (interven*[Title/Abstract]) | | | "pilot"[Title/Abstract] AND "obes*"[Title/Abstract] AND "fitness"[Title/Abstract] AND "interven*"[Title/Abstract] |
| 28 | (((feasibility[Title/Abstract]) AND (obes*[Title/Abstract])) AND (fitness[Title/Abstract])) AND (interven*[Title/Abstract]) | | | "feasibility"[Title/Abstract] AND "obes*"[Title/Abstract] AND "fitness"[Title/Abstract] AND "interven*"[Title/Abstract] |
| 29 | (((feasibility[Title/Abstract]) AND (obes*[Title/Abstract])) AND (fitness[Title/Abstract])) AND (interven*[Title/Abstract]) | | from 1995 - 2020 | ("feasibility"[Title/Abstract] AND "obes*"[Title/Abstract] AND "fitness"[Title/Abstract] AND "interven*"[Title/Abstract]) AND (1995:2020[pdat]) |
| 27 | (((pilot[Title/Abstract]) AND (obes*[Title/Abstract])) AND (sedentary[Title/Abstract])) AND (interven*[Title/Abstract]) | | from 2001 - 2020 | ("pilot"[Title/Abstract] AND "obes*"[Title/Abstract] AND "sedentary"[Title/Abstract] AND "interven*"[Title/Abstract]) AND (2001:2020[pdat]) |
| 26 | (((pilot[Title/Abstract]) AND (obes*[Title/Abstract])) AND (sedentary[Title/Abstract])) AND (interven*[Title/Abstract]) | | | "pilot"[Title/Abstract] AND "obes*"[Title/Abstract] AND "sedentary"[Title/Abstract] AND "interven*"[Title/Abstract] |
| 25 | (((feasibility[Title/Abstract]) AND (obes*[Title/Abstract])) AND (sedentary[Title/Abstract])) AND (interven*[Title/Abstract]) | | from 1998 - 2020 | ("feasibility"[Title/Abstract] AND "obes*"[Title/Abstract] AND "sedentary"[Title/Abstract] AND "interven*"[Title/Abstract]) AND (1998:2020[pdat]) |
| 24 | (((feasibility[Title/Abstract]) AND (obes*[Title/Abstract])) AND (sedentary[Title/Abstract])) AND (interven*[Title/Abstract]) | | | "feasibility"[Title/Abstract] AND "obes*"[Title/Abstract] AND "sedentary"[Title/Abstract] AND "interven*"[Title/Abstract] |
| 23 | (((pilot[Title/Abstract]) AND (obes*[Title/Abstract])) AND (nutrition[Title/Abstract])) AND (interven*[Title/Abstract]) | | from 1998 - 2020 | ("pilot"[Title/Abstract] AND "obes*"[Title/Abstract] AND "nutrition"[Title/Abstract] AND "interven*"[Title/Abstract]) AND (1998:2020[pdat]) |
| 22 | (((pilot[Title/Abstract]) AND (obes*[Title/Abstract])) AND (nutrition[Title/Abstract])) AND (interven*[Title/Abstract]) | | | "pilot"[Title/Abstract] AND "obes*"[Title/Abstract] AND "nutrition"[Title/Abstract] AND "interven*"[Title/Abstract] |
| 21 | (((feasibility[Title/Abstract]) AND (obes*[Title/Abstract])) AND (nutrition[Title/Abstract])) AND (interven*[Title/Abstract]) | | from 1994 - 2020 | ("feasibility"[Title/Abstract] AND "obes*"[Title/Abstract] AND "nutrition"[Title/Abstract] AND "interven*"[Title/Abstract]) AND (1994:2020[pdat]) |
| 20 | (((feasibility[Title/Abstract]) AND (obes*[Title/Abstract])) AND (nutrition[Title/Abstract])) AND (interven*[Title/Abstract]) | | | "feasibility"[Title/Abstract] AND "obes*"[Title/Abstract] AND "nutrition"[Title/Abstract] AND "interven*"[Title/Abstract] |
| 19 | (((pilot[Title/Abstract]) AND (obes*[Title/Abstract])) AND (diet[Title/Abstract])) AND (interven*[Title/Abstract]) | | from 1996 - 2020 | ("pilot"[Title/Abstract] AND "obes*"[Title/Abstract] AND "diet"[Title/Abstract] AND "interven*"[Title/Abstract]) AND (1996:2020[pdat]) |
| 18 | (((pilot[Title/Abstract]) AND (obes*[Title/Abstract])) AND (diet[Title/Abstract])) AND (interven*[Title/Abstract]) | | | "pilot"[Title/Abstract] AND "obes*"[Title/Abstract] AND "diet"[Title/Abstract] AND "interven*"[Title/Abstract] |
| 17 | (((feasibility[Title/Abstract]) AND (obes*[Title/Abstract])) AND (diet[Title/Abstract])) AND (interven*[Title/Abstract]) | | from 1976 - 2020 | ("feasibility"[Title/Abstract] AND "obes*"[Title/Abstract] AND "diet"[Title/Abstract] AND "interven*"[Title/Abstract]) AND (1976:2020[pdat]) |
| 16 | (((feasibility[Title/Abstract]) AND (obes*[Title/Abstract])) AND (diet[Title/Abstract])) AND (interven*[Title/Abstract]) | | | "feasibility"[Title/Abstract] AND "obes*"[Title/Abstract] AND "diet"[Title/Abstract] AND "interven*"[Title/Abstract] |
| 15 | (((pilot[Title/Abstract]) AND (obes*[Title/Abstract])) AND (exercise[Title/Abstract])) AND (interven*[Title/Abstract]) | | from 1996 - 2020 | ("pilot"[Title/Abstract] AND "obes*"[Title/Abstract] AND "exercise"[Title/Abstract] AND "interven*"[Title/Abstract]) AND (1996:2020[pdat]) |
| 14 | (((pilot[Title/Abstract]) AND (obes*[Title/Abstract])) AND (exercise[Title/Abstract])) AND (interven*[Title/Abstract]) | | | "pilot"[Title/Abstract] AND "obes*"[Title/Abstract] AND "exercise"[Title/Abstract] AND "interven*"[Title/Abstract] |
| 13 | (((feasibility[Title/Abstract]) AND (obes*[Title/Abstract])) AND (exercise[Title/Abstract])) AND (interven*[Title/Abstract]) | | from 1995 - 2020 | ("feasibility"[Title/Abstract] AND "obes*"[Title/Abstract] AND "exercise"[Title/Abstract] AND "interven*"[Title/Abstract]) AND (1995:2020[pdat]) |
| 12 | (((feasibility[Title/Abstract]) AND (obes*[Title/Abstract])) AND (exercise[Title/Abstract])) AND (interven*[Title/Abstract]) | | | "feasibility"[Title/Abstract] AND "obes*"[Title/Abstract] AND "exercise"[Title/Abstract] AND "interven*"[Title/Abstract] |
| 11 | (((pilot[Title/Abstract]) AND (obes*[Title/Abstract])) AND ("physical activity"[Title/Abstract])) AND (interven*[Title/Abstract]) | | from 1981 - 2020 | ("pilot"[Title/Abstract] AND "obes*"[Title/Abstract] AND "physical activity"[Title/Abstract] AND "interven*"[Title/Abstract]) AND (1981:2020[pdat]) |
| 10 | (((pilot[Title/Abstract]) AND (obes*[Title/Abstract])) AND ("physical activity"[Title/Abstract])) AND (interven*[Title/Abstract]) | | | "pilot"[Title/Abstract] AND "obes*"[Title/Abstract] AND "physical activity"[Title/Abstract] AND "interven*"[Title/Abstract] |
| 9 | (((feasibility[Title/Abstract]) AND (obes*[Title/Abstract])) AND ("physical activity"[Title/Abstract])) AND (interven*[Title/Abstract]) | | from 1989 - 2020 | ("feasibility"[Title/Abstract] AND "obes*"[Title/Abstract] AND "physical activity"[Title/Abstract] AND "interven*"[Title/Abstract]) AND (1989:2020[pdat]) |
| 8 | (((feasibility[Title/Abstract]) AND (obes*[Title/Abstract])) AND ("physical activity"[Title/Abstract])) AND (interven*[Title/Abstract]) | | | "feasibility"[Title/Abstract] AND "obes*"[Title/Abstract] AND "physical activity"[Title/Abstract] AND "interven*"[Title/Abstract] |
| 7 | (((feasibility) AND (obes*)) AND ("physical activity")) AND (interven*) | | from 1980 - 2020 | (("feasibilities"[All Fields] OR "feasibility"[All Fields] OR "feasible"[All Fields] OR "feasiblity"[All Fields]) AND "obes*"[All Fields] AND "physical activity"[All Fields] AND "interven*"[All Fields]) AND (1980:2020[pdat]) |
| 6 | (((feasibility) AND (obes*)) AND ("physical activity")) AND (interven*) | | | ("feasibilities"[All Fields] OR "feasibility"[All Fields] OR "feasible"[All Fields] OR "feasiblity"[All Fields]) AND "obes*"[All Fields] AND "physical activity"[All Fields] AND "interven*"[All Fields] |
| 5 | ((pilot[Title/Abstract]) AND (obes*[Title/Abstract])) AND (interven*[Title/Abstract]) | | from 1981 - 2020 | ("pilot"[Title/Abstract] AND "obes*"[Title/Abstract] AND "interven*"[Title/Abstract]) AND (1981:2020[pdat]) |
| 4 | ((pilot[Title/Abstract]) AND (obes*[Title/Abstract])) AND (interven*[Title/Abstract]) | | | "pilot"[Title/Abstract] AND "obes*"[Title/Abstract] AND "interven*"[Title/Abstract] |
| 3 | ((feasibility[Title/Abstract]) AND (obes*[Title/Abstract])) AND (interven*[Title/Abstract]) | | from 1976 - 2020 | ("feasibility"[Title/Abstract] AND "obes*"[Title/Abstract] AND "interven*"[Title/Abstract]) AND (1976:2020[pdat]) |
| 2 | ((feasibility[Title/Abstract]) AND (obes*[Title/Abstract])) AND (interven*[Title/Abstract]) | | | "feasibility"[Title/Abstract] AND "obes*"[Title/Abstract] AND "interven*"[Title/Abstract] |
| 1 | (((feasibility[Title/Abstract]) AND (obes*[Title/Abstract])) AND (interven*[Title/Abstract])) AND ("2017/01/01"[Date - Publication] : "2020/12/31"[Date - Publication]) | | | "feasibility"[Title/Abstract] AND "obes*"[Title/Abstract] AND "interven*"[Title/Abstract] AND 2017/01/01:2020/12/31[Date - Publication] |
